# Loss of the collagen IV modifier prolyl 3-hydroxylase 2 causes thin basement membrane nephropathy

**DOI:** 10.1172/JCI147253

**Published:** 2022-05-02

**Authors:** Hande Aypek, Christoph Krisp, Shun Lu, Shuya Liu, Dominik Kylies, Oliver Kretz, Guochao Wu, Manuela Moritz, Kerstin Amann, Kerstin Benz, Ping Tong, Zheng-mao Hu, Sulaiman M. Alsulaiman, Arif O. Khan, Maik Grohmann, Timo Wagner, Janina Müller-Deile, Hartmut Schlüter, Victor G. Puelles, Carsten Bergmann, Tobias B. Huber, Florian Grahammer

**Affiliations:** 1 III. Department of Medicine and; 2 Institute of Clinical Chemistry and Laboratory Medicine, Mass Spectrometric Proteomics Group, University Medical Center Hamburg-Eppendorf, Hamburg, Germany.; 3 Department of Nephropathology, Institute of Pathology and; 4 Department of Pediatrics, University of Erlangen, Erlangen, Germany.; 5 Department of Ophthalmology, The Second Xiangya Hospital and; 6 Center for Medical Genetics, School of Life Sciences, Central South University, Changsha, Hunan, China.; 7 Vitreoretinal Division, King Khaled Eye Specialist Hospital, Riyadh, Saudi Arabia.; 8 Eye Institute, Cleveland Clinic Abu Dhabi, Abu Dhabi, United Arab Emirates.; 9 Department of Ophthalmology, Cleveland Clinic Lerner College of Medicine of Case Western University, Cleveland, Ohio, USA.; 10 Medizinische Genetik Mainz, Limbach Genetics, Mainz, Germany.; 11 Department of Nephrology, Friedrich-Alexander-Universität Erlangen-Nürnberg, Erlangen, Germany.; 12 Department of Medicine IV, Faculty of Medicine, Medical Center-University of Freiburg, Freiburg, Germany.

**Keywords:** Nephrology, Chronic kidney disease, Collagens, Monogenic diseases

## Abstract

The glomerular filtration barrier (GFB) produces primary urine and is composed of a fenestrated endothelium, a glomerular basement membrane (GBM), podocytes, and a slit diaphragm. Impairment of the GFB leads to albuminuria and microhematuria. The GBM is generated via secreted proteins from both endothelial cells and podocytes and is supposed to majorly contribute to filtration selectivity. While genetic mutations or variations of GBM components have been recently proposed to be a common cause of glomerular diseases, pathways modifying and stabilizing the GBM remain incompletely understood. Here, we identified prolyl 3-hydroxylase 2 (*P3H2*) as a regulator of the GBM in an a cohort of patients with albuminuria. P3H2 hydroxylates the 3′ of prolines in collagen IV subchains in the endoplasmic reticulum. Characterization of a *P3h2^ΔPod^* mouse line revealed that the absence of P3H2 protein in podocytes induced a thin basement membrane nephropathy (TBMN) phenotype with a thinner GBM than that in WT mice and the development of microhematuria and microalbuminuria over time. Mechanistically, differential quantitative proteomics of the GBM identified a significant decrease in the abundance of collagen IV subchains and their interaction partners in *P3h2^ΔPod^* mice. To our knowledge, P3H2 protein is the first identified GBM modifier, and loss or mutation of *P3H2* causes TBMN and focal segmental glomerulosclerosis in mice and humans.

## Graphical abstract

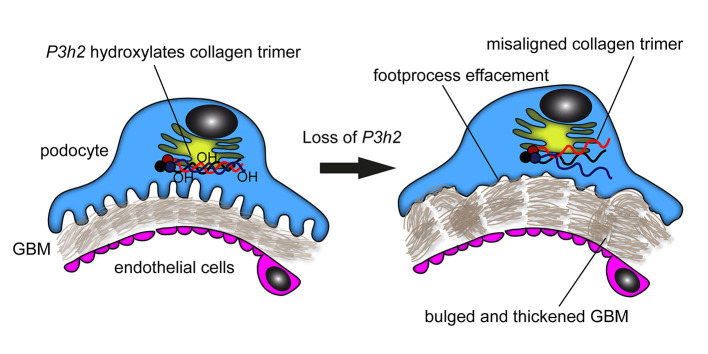

## Introduction

Renal filtration is a highly regulated process leading to the formation of cell-free and virtually albumin-free primary urine. Over the past 2 decades, more than 50 monogenic causes of malfunction of the glomerular filtration barrier (GFB) have been described, most due to mutations in genes highly expressed in podocytes (1). One subgroup comprises defects in the formation of the glomerular basement membrane (GBM). The GBM is the central meshwork structure of the GFB. Kidney diseases related to mutations in *COL4A1*, *COL4A3*, *COL4A4*, and *COL4A5* are called collagen IV nephropathies. Alport syndrome and thin basement membrane nephropathy (TBMN) are 2 prominent examples of collagen IV nephropathies (2). Moreover, variants in the above *COL4* genes are the most commonly identified gene mutations for adult-onset focal segmental glomerulosclerosis (FSGS) (3, 4). Alport syndrome is, and TBMN can be, a progressive glomerular disease, and over decades patients may develop FSGS and end-stage kidney disease (5–7). The prevalence of TBMN in the general population is difficult to estimate, as many patients are not aware of their condition (8). Patients with early Alport syndrome or TBMN have similar urinary signs, i.e., hematuria and microalbuminuria. However, the GBM phenotype appears different already at early stages: patients with Alport syndrome have a lamellated GBM, whereas patients with TBMN have a thinner GBM (8, 9).

Collagen IV is the major structural GBM protein and has 6 different α-chains forming isomer-specific trimers (α 1α 1α 2, α3α4α5, and α55α6) (10). Collagen IV α3α4α5 is the most abundant trimer in adult GBM and mostly secreted via podocytes (11). Proper collagen IV secretion and cross-linking are important for GBM homeostasis. Major functions of the GBM include filtrating blood, providing a scaffold for resident glomerular cells, and enabling crosstalk between podocytes and endothelial cells (12). Disruption of GBM homeostasis results in structural damage to the GFB, with subsequent development and progression of kidney failure (13). Therefore, detailed investigations into homeostatic mechanisms within the GBM are necessary to better understand the mechanism of glomerular ultrafiltration.

The enzyme prolyl 3-hydroxylase 2 (P3H2) is a posttranslational modifier and catalyzes 3′ hydroxylation (3Hyp) of proline residues of collagen IV. Proline is often found in position 2 or 3 of the Gly-Xaa-Yaa consensus repeats in the intermediate collagenous domain, and hydroxylation confers flexibility. P3H2 is localized in the endoplasmic reticulum–Golgi network and is highly expressed in tissues that have a rich basement membrane such as those of the kidney and eye (14). Mutations in the *P3H2* gene can result in high myopia, lens subluxation, cataract formation, and a predisposition to retinal detachment (15). The exact number and localization of 3Hyp sites of collagen IV subchains are not completely understood to date. Although their effect on collagen IV structure and function remains unclear, it is hypothesized that 3Hyp of collagen IV might have a role in chain assembly, crosslinking, stability, and protein-protein interaction (16).

We identified what we believe to be a new albuminuria candidate gene, *P3H2*, using an expression-based approach in a cohort of patients with albuminuria. The relevance of P3H2-mediated collagen modifications for GBM function were not known. We therefore explored the potential effects of P3H2 loss-of-function mutations on collagen IV synthesis and GBM homeostasis in vitro and in vivo. Our study shows that podocyte-specific *P3h2*-KO mice (referred to hereafter as *P3h2^ΔPod^* or KO mice) mice initially have a TBMN phenotype that slowly progresses to a FSGS phenotype. These findings in mice were supported by data from 3 human kindreds with *P3H2* loss-of-function mutations and ocular defects. In conclusion, we identify the what we believe to be the first nonstructural GBM modifier and prove its relevance for glomerular homeostasis.

## Results

### Identification of P3H2 as a candidate gene in a cohort of patients with albuminuria.

**Figure 1:**
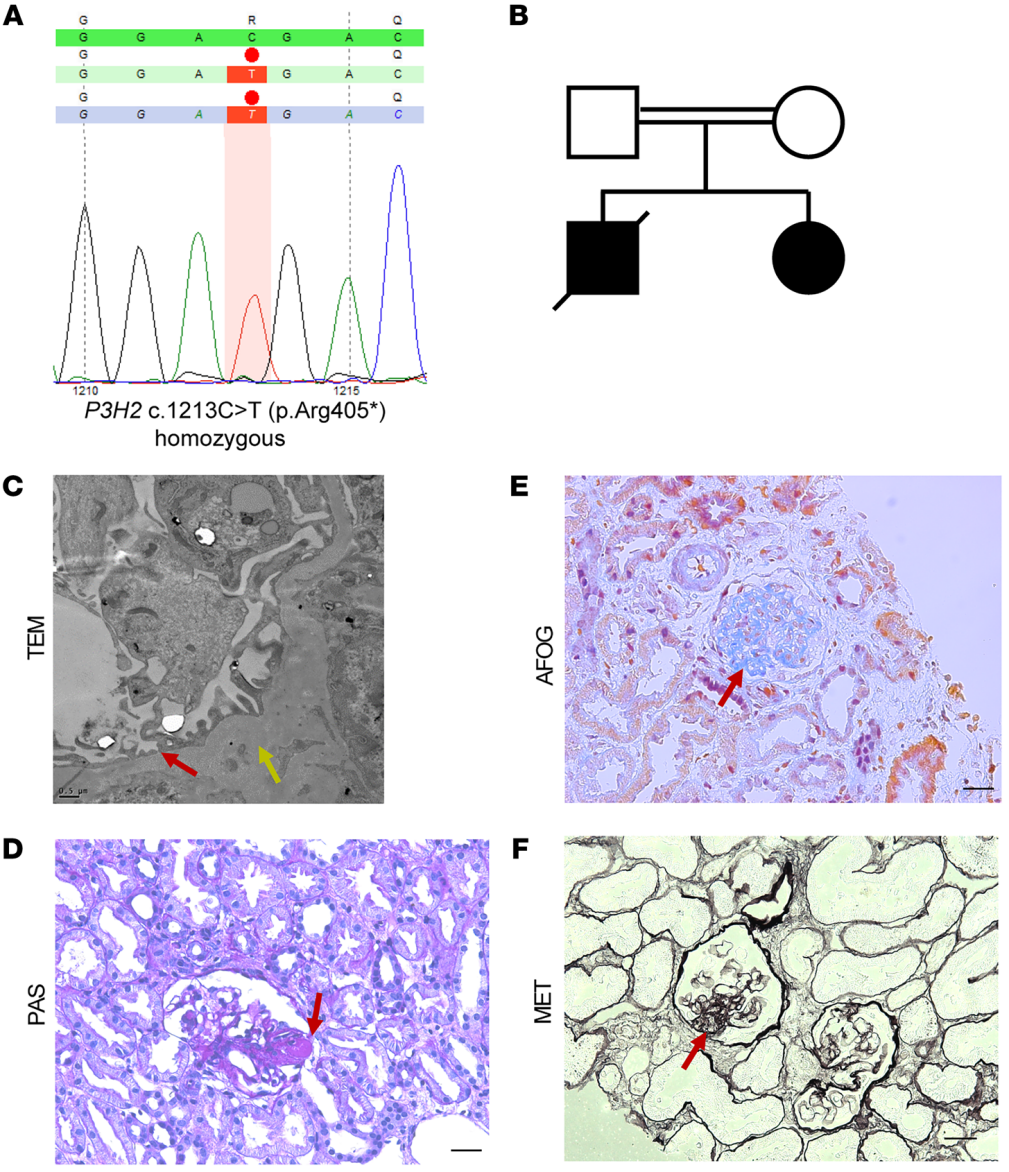
Histologic examination of a kidney biopsy from a patient with a *P3H2* gene mutation, in a cohort of patients with albuminuria. (**A**) Exact position of the *P3H2* mutation. A nonsense mutation was detected at exon 4, with transition from cytosine (C) to thymidine (T) at position 1213, leading to a premature stop codon. (**B**) Pedigree of the patient. The patient was the second child of consanguineous parents, who was diagnosed with FSGS. Her brother was also diagnosed with FSGS but died during childhood. (**C**) Foot process effacement (red arrow) and an irregular and thickened GBM (yellow arrow) was observed on TEM micrographs of the patient’s biopsy. Scale bar: 0.5 μm. (**D**) Representative PAS staining showed focal glomerulosclerosis (red arrow). (**E**) Fibrosis was observed on AFOG staining (red arrow). (**F**) Representative MET staining shows a thickened GBM (red arrow). Scale bars: 20 μm (**D**–**F**).

Potential candidate genes were detected in an albuminuric patient cohort via correlation of highly expressed mouse podocyte genes with human expression data sets (17). We scored the identified candidate genes using this criterion as well as enrichment in respective gene ontology (GO) terms. The *P3H2* gene was identified in a mouse and human glomerular expression database and highly rated in GO terms related to matrix biology. Subsequently, we identified a homozygous pathogenic nonsense mutation in a young female patient with steroid-resistant nephrotic syndrome (SRNS) and high myopia and cataracts. The *P3H2* mutation at exon 4 caused a transition from cytosine (C) to thymidine (T) at position 1213, inducing the formation of a premature stop codon (c.1213C>T, p.Arg405Ter), while no other pathogenic variant of causative FSGS/SRNS genes could be detected (Figure 1A). In the family’s pedigree, she was the second child of a consanguineous marriage and had a brother with a known diagnosis of FSGS, who died at the age of 6 (Figure 1B).

Histological examination of the kidney biopsy tissue was done using standard histochemistry staining techniques. We observed glomerular damage and partial glomerulosclerosis in periodic acid–Schiff (PAS) staining of the patient’s biopsy (Figure 1D). Fibrosis and GBM thickening of the glomeruli were detected in acid fuchsin orange G (AFOG) and methenamine silver (MET) staining, respectively (Figure 1, E and F). We also examined sections of kidney biopsy tissue from the patient by transition electron microscopy (TEM) and observed foot process effacement of podocytes and GBM irregularities (thickening and thinning) in the micrographs (Figure 1C). These data and clinical follow-up showed that our patient had progressive FSGS that ultimately proceeded to end-stage kidney disease.

Next, we analyzed patients with ocular abnormalities (e.g., myopia, cataract, lens luxation) related to *P3H2* mutations for urinary abnormalities. We analyzed the urine of 2 unrelated pedigrees. In the first family, there were 2 siblings carrying a homozygous *P3H2* mutation in exon1 (c.13C>T; p.Q5X), inducing the formation of a premature stop codon. Both of these siblings had high myopia and cataract (15). Their urinalysis showed microhematuria. In the second family, there were 4 siblings aged between 11 and 21 years, who presented with high myopia, retinal detachment, and cataract. Each of them was found to be homozygous for a *P3H2* frameshift mutation (c.679G>T; p.Glu277*). Their ocular phenotype resembled that of a previously reported family from the same region with a different homozygous mutation in the gene (18). Urinalysis of these patients showed that all but 1 of the 4 siblings had microalbuminuria and microhematuria (Supplemental Figure 1; supplemental material available online with this article; https://doi.org/10.1172/JCI147253DS1). These data from a total of 3 unrelated ancestries harboring different mutations highly suggest the modulating effects of P3H2 on the GBM in humans.

We evaluated the expression of P3H2 at both the mRNA and protein levels in mouse and human glomeruli and podocytes. Although *P3h2* mRNA was not detected in mouse embryonic tissue at E14.5 by ISH (Supplemental Figure 2A), glomerular localization of *P3h2* mRNA could be detected in P1 mouse kidney (Supplemental Figure 2B). The ISH data underline that *P3h2* expression coincides with glomerular maturation. We observed colocalization of P3H2 with NPHS1, a podocyte marker, in immunofluorescence stainings of both human and mouse kidney tissues (Supplemental Figure 2C). In quantitative PCR (qPCR) of sorted mouse glomerular cells, the relative mRNA expression of *P3h2* was 75.7% ± 18.5% in podocytes, 0.05% ± 0.02% in glomerular endothelial cells, and 7.9% ± 6.1% in mesangial cells (Supplemental Figure 2D). This showed that podocytes had the highest mRNA level of *P3h2* in comparison with glomerular endothelial and mesangial cells. Using Western blotting, we could detect P3H2 protein in all samples, with the highest relative abundance in immortalized human podocytes (Supplemental Figure 2E). Taken together, we validated that podocytes are the main glomerular cells expressing *P3H2* and that patients harboring *P3H2* mutations have urinary abnormalities.

### Podocyte-specific P3h2 loss results in TBMN.

**Figure 2:**
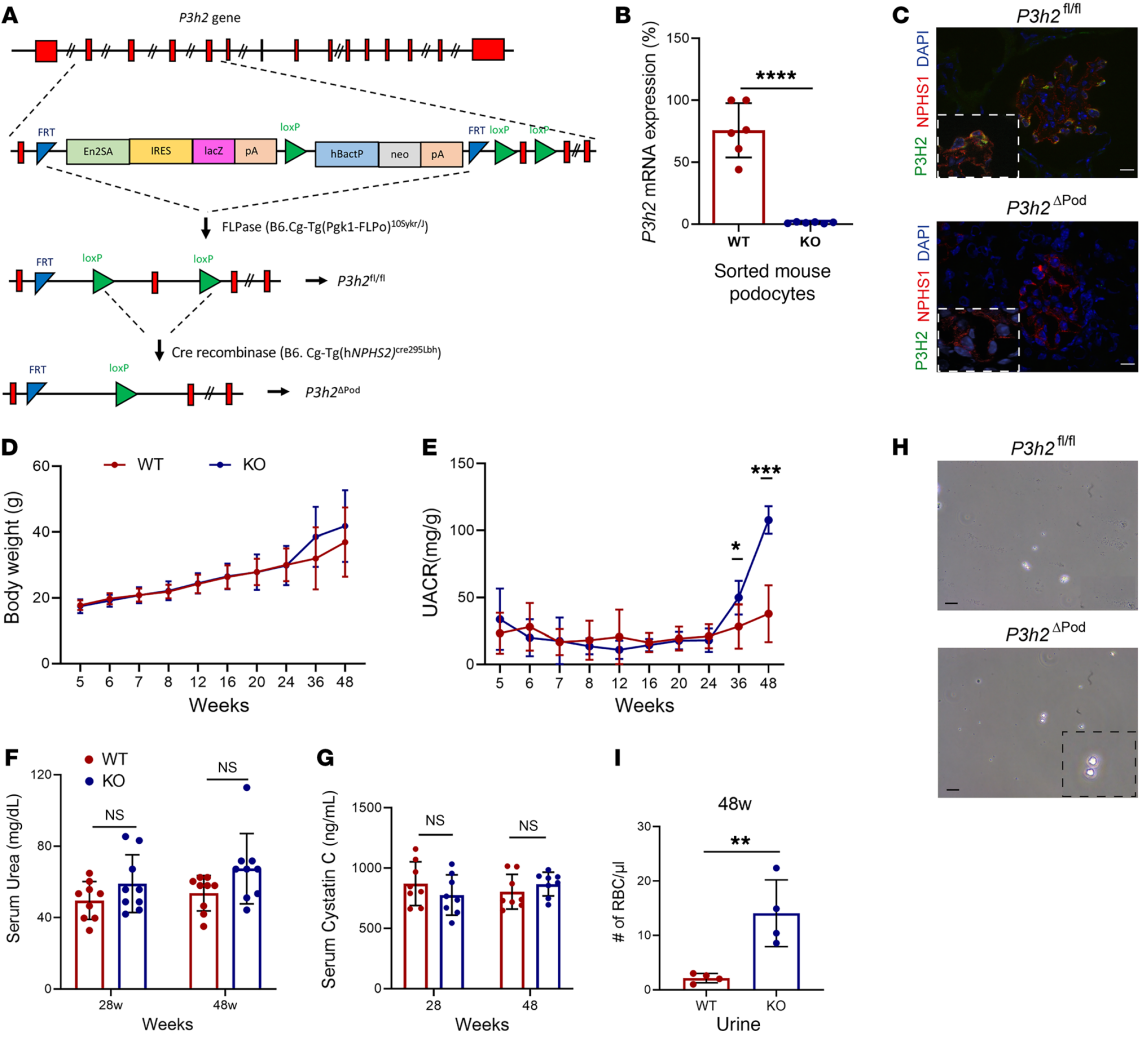
Generation and characterization of *P3h2^ΔPod^* mice by urinalysis. (**A**) Schematic strategy for the generation of *P3h2^ΔPod^* mice. (**B**) KO confirmation of the *P3h2^ΔPod^* mice. qPCR *P3h2* mRNA expression analysis was performed in sorted podocytes from WT and KO mice. *P3h2* mRNA levels were reduced by 99% in *P3h2^ΔPod^* mice compare with levels in *P3h2^fl/fl^* mice. (**C**) Immunofluorescence staining of WT and KO kidney tissues for P3H2, NPHS1, and DAPI. There was no detectable P3H2 in the *P3h2^ΔPod^* mouse podocytes. Scale bar: 10 μm; inset zoom, ×5. (**D**) Body weights of *P3h2^ΔPod^* and *P3h2^fl/fl^* mice were measured starting at 5 weeks until 48 weeks of age. There was no significant difference at any time point in body weights between the WT and KO mice (**E**) UACR of *P3h2^ΔPod^* and *P3h2^fl/fl^* mice. KO mice started to present with albuminuria at 36 weeks of age, and this had increased at 48 weeks of age. (**F**) Serum urea measurements for *P3h2^ΔPod^* and *P3h2^fl/fl^* mice. No significant increase was observed in the KO mice. (**G**) Serum cystatin C measurement for *P3h2^ΔPod^* and *P3h2^fl/fl^* mice. No significant increase was observed in the KO mice. (**H**) Hematuria was detected in spot urine of *P3h2^ΔPod^* mice. Representative images of urine from mice of each genotype. There were significantly more and dysmorphic RBCs in KO urine than WT urine. Scale bar: 50 μm: inset zoom, ×5. (**I**) Quantification of urinary RBCs under a light microscope. *n* ≥ 3. Graphs show the mean ± SD. *P < 0.05, **P < 0.01, ***P < 0.001, and ****P < 0.0001, by unpaired, 2-tailed Student’s *t* test.

We generated podocyte-specific *P3h2*-KO (*P3h2^ΔPod^*) mice to study the effect of P3H2 on GBM and GFB homeostasis. As indicated in Figure 2A, floxed allele *P3h2* (*P3h2^fl/fl^*) mice were generated by breeding *P3h2^tm1aWtsi^* mice with *FLPase* mice. Subsequently, *P3h2^fl/fl^* mice were bred with *hNphs2Cre^WT/+^* mice to specifically delete the *P3h2* gene in podocytes. *P3h2^fl/fl^* mice without Cre recombinase were used as WT controls in all experiments. We chose 3 time points (6 weeks, 28 weeks, and 48 weeks) for further in-depth characterization of the *P3h2^ΔPod^* mouse line. A lack of *P3h2* expression in podocytes of the generated mouse line was proven by qPCR and immunofluorescence staining. In qPCR of sorted podocytes, the relative mRNA expression of *P3h2* in WT podocytes was 75.7% ± 18.5% and 1.17% ± 0.48% in KO podocytes (*P <* 0.0001; Figure 2B), indicating that *P3h2* mRNA levels were dramatically decreased in the podocytes of KO mice compared with levels in WT podocytes. Moreover, using immunofluorescence staining, we could not detect P3H2 protein expression in podocytes in the KO mice (Figure 2C). Both qPCR and immunofluorescence staining data demonstrated that the generated *P3h2^ΔPod^* mouse line lacked *P3h2* expression in podocytes.

The body weights of KO and WT animals were close to each other at every time point over the measurement period, and the differences at 36 weeks and 48 weeks were not significant (Figure 2D). Urinalysis was our first step in characterizing *P3h2^ΔPod^* mice to observe functional signs of glomerular disease development. Urinary albumin/creatinine ratio (UACR) analysis shows that until the age of 36 weeks, KO mice did not have albuminuria. At approximately 36 weeks, KO mice started to leak albumin 50.1 ± 12.7 mg/g (*n =* 8), whereas albumin in WT mice was 28.4 ± 16.4 mg/g (*n =* 8). Albuminuria further increased by 48 weeks, measuring 107.7 ± 10.3 mg/g (*n =* 8) in the KO mice (*P <* 0.001; Figure 2E). Serum urea was 49.6 ± 10.6 mg/dL (*n =* 8) for WT mice and 58.9 ± 16.3 mg/dL (*n =* 8) for KO mice at 28 weeks. For 48-week-old mice, WT mice had 53.7 ± 9.9 ng/dL (*n =* 8) and KO mice had 67.4 ± 19.7 mg/dL (*n =* 8) serum urea (*P >* 0.05; Figure 2F and Supplemental Figure 3). Serum cystatin C values were 871.1 ± 181.2 ng/mL (*n =* 8) for WT mice and 777.2 ± 167.6 ng/mL (*n =* 8) for KO mice at 28 weeks. For 48-week-old mice, WT mice had 803.3 ± 144.5 ng/mL (*n =* 8) and KO mice had 867.4 ± 98.5 ng/mL (*n =* 8) cystatin C in serum. These values were within the normal range and were not significantly different between WT and KO mice at the age of 28 weeks and 48 weeks, respectively (*P >* 0.05; Figure 2G). We tested for microhematuria and detected dysmorphic RBCs in the urine of the KO mice. There were 14 ± 7 cells per μL (*n =* 4) in KO mouse urine and only 2 ± 1 cells per μL in WT mouse urine (*n =* 4) (*P <* 0.01; Figure 2, H and I). Microhematuria, which we observed in the *P3h2^ΔPod^* mice, represents a common sign of collagen IV nephropathies. In summary, urinalysis indicated the development of progressive glomerular damage in the *P3h2^ΔPod^* mice.

**Figure 3:**
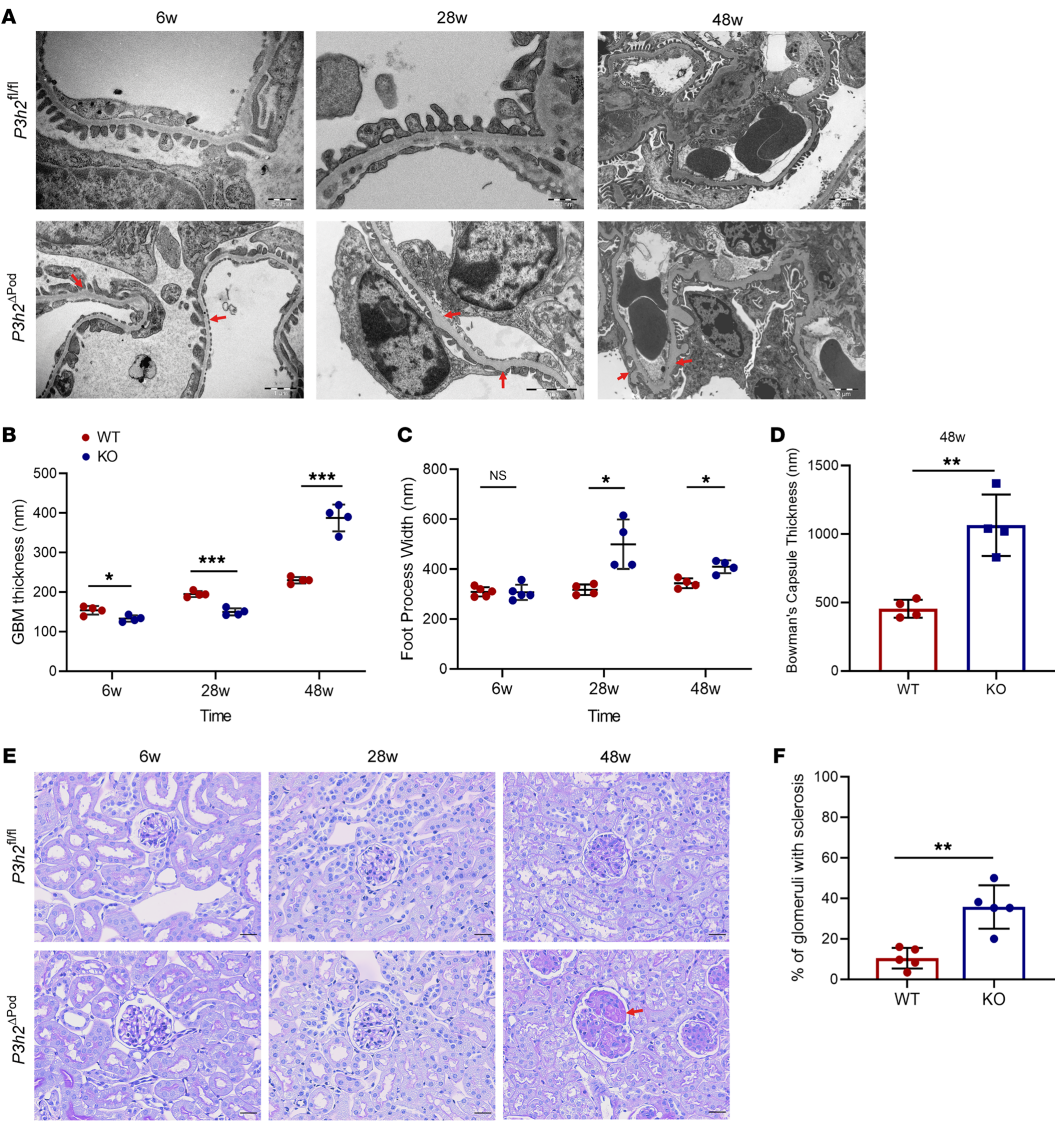
Ultrastructural analysis and histological phenotypes of *P3h2^ΔPod^* and *P3h2^fl/fl^* mice. (**A**) TEM micrographs of *P3h2^ΔPod^* and *P3h2^fl/fl^* mice at 6 weeks, 28 weeks, and 48 weeks. Red arrows indicate an abnormal GBM structure. Scale bars: 500 nm (top left and middle panels), 2 μm (top right panel), 1 μm (bottom left panel), and 2 μm (bottom middle and right panels). (**B**) Measurement of GBM thickness on TEM micrographs. At 6 weeks and 28 weeks, the KO mice had a thinner GBM, whereas at 48 weeks, the KO mice had a thicker GBM when compared with that of WT mice. (**C**) Foot process width measurement of WT and KO podocytes on TEM micrographs. Foot process widths were increased in 28- and 48-week-old KO mice when compared with WT foot process widths, indicating foot process effacement. (**D**) Bowman’s capsule thickness measurement on TEM micrographs of *P3h2^ΔPod^* and *P3h2^fl/fl^* mice at 48 weeks. The thickness of Bowman’s capsule was significantly increased in KO mice, indicating PEC activation. (**E**) PAS staining was performed at 6 weeks, 28 weeks, and 48 weeks. At 6 weeks and 28 weeks, the glomerular morphology was healthy in *P3h2^ΔPod^* mice. At 48 weeks, glomerulosclerosis and podocyte injury were observed in *P3h2^ΔPod^* kidney tissue (red arrow). Scale bar: 20 μm. (**F**) Quantification of glomerulosclerosis in *P3h2^ΔPod^* and *P3h2^fl/fl^* mice at 48 weeks. *P3h2^ΔPod^* mice had significantly more glomerulosclerosis than did *P3h2^fl/fl^* mice. *n* ≥4. Graphs show the mean ± SD. *P < 0.05, **P < 0.01, and ***P < 0.001, by unpaired, 2-tailed Student’s *t* test.

We performed ultrastructural and histological evaluation of *P3h2^ΔPod^* mice kidneys using TEM and PAS staining, respectively. Although with PAS staining we detected no apparent difference from 6 weeks to 28 weeks, TEM indicated overall thinning of the GBM, with small areas showing a focal, loose arrangement of GBM constituents appearing as slightly thickened areas. We observed foot process effacement, thickening of the GBM and Bowman’s capsule, as well as glomerulosclerosis in glomeruli of *P3h2^ΔPod^* mice at 48 weeks (Figure 3A). At 6 weeks, the thickness of the GBM for *P3h2^ΔPod^* mice was 124.7± 12.8 nm (*n =* 4), while that of *P3h2^fl/fl^* mice was 154.1 ± 15.3 nm (*n =* 4) (*P <* 0.05). At 28 weeks, it was 149.9 ± 9.1 nm (*n =* 4) for *P3h2^ΔPod^* and was 195.2 ± 7.5 nm for *P3h2^fl/fl^* mice (*n =* 4) (*P <* 0.001). At 48 weeks, the thickness of the GBM for *P3h2^ΔPod^* mice was 387.5 ± 34 nm (*n =* 4), and for *P3h2^fl/fl^* mice, it was 230 ± 8.1 nm (*n =* 4) (*P <* 0.001) (Figure 3B). Quantification of the TEM micrographs revealed that the KO mice had a thinner GBM than did the WT mice at 6 weeks and 28 weeks, similar to early signs of TBMN in humans. At 48 weeks, the thickness of the GBM increased significantly as a result of glomerulosclerosis. In addition, quantification of foot process widths indicated that there was no difference at 6 weeks between KO and WT mice. The mean foot process width was 311.9 ± 35.5 nm (*n =* 4) for KO mice and 310.8 ± 21.9 nm (*n =* 4) (*P >* 0.05) for WT mice. At 28 weeks, the mean foot process width was 499.9 ± 98.9 nm (*n =* 4) for KO mice and 317.7 ± 21.5 nm (*n =* 4) (*P <* 0.05) for WT mice. At 48 weeks, the mean values of the foot process width for KO mice were 409.9 ± 25.5 nm (*n =* 4) and for WT mice, 343.7 ± 19.2 nm (*n =* 4) (*P <* 0.05; Figure 3C). Foot process widths were significantly greater in KO mice at 28 weeks and 48 weeks, indicating foot process effacement. We quantified the Bowman’s capsule thickness at 48 weeks (KO mice: 1065 ± 224.2 nm, *n =* 4; WT mice: 455 ± 66.1 nm, *n =* 4; *P <* 0.01) (Figure 3D).

We observed a healthy glomerular appearance in *P3h2^ΔPod^* mice at 6 weeks and 28 weeks with PAS staining. At 48 weeks, glomerulosclerosis was observed and quantified in *P3h2^ΔPod^* glomeruli, which showed further signs of podocyte injury. In *P3h2^fl/fl^* mice, the glomeruli appeared healthy at this time point (Figure 3E). The percentage of glomerulosclerosis in KO glomeruli was 35.8% ± 10.6% (*n =* 5) and 10.4% ± 5.1% in WT glomeruli (*n =* 5) (*P <* 0.01). The number of glomeruli showing focal glomerulosclerosis was significantly higher in KO mice than in WT mice, confirming the FSGS phenotype in the KO mice (Figure 3F). In summary, these data prove that *P3h2* deletion affects the GBM as early as 6 weeks and that the mice developed a more severe phenotype at later stages of life, indicating progressive glomerular disease.

### Proper collagen IV network formation is lacking in the GBM of P3h2^ΔPod^ mice.

**Figure 4:**
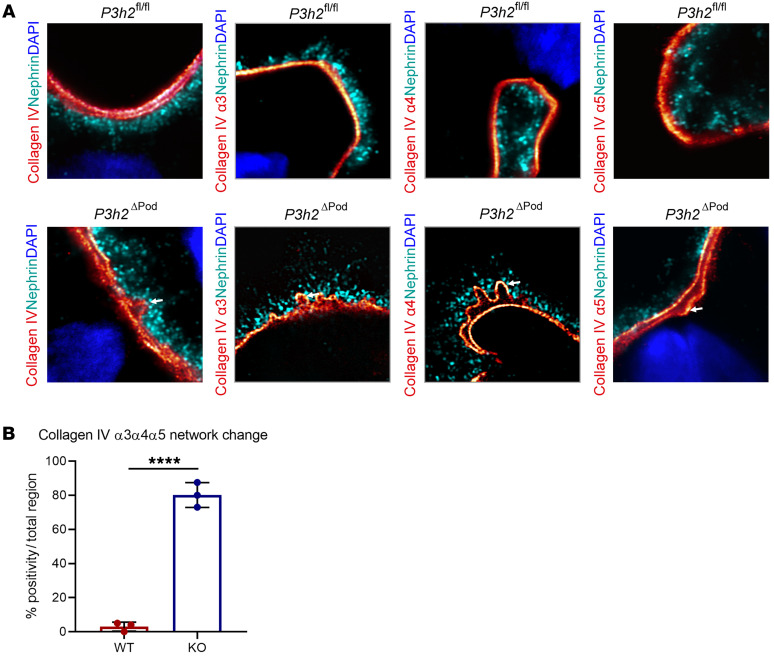
High-resolution imaging to visualize collagen IV α3α4α5 network formation. (**A**) High-resolution imaging of glomeruli from *P3h2^ΔPod^* and *P3h2^fl/fl^* mice with expansion microscopy. In WT GBM, a linear collagen IV localization was observed for collagen IV and collagen IV α3, α4, and α5 proteins, indicating proper network formation. However, in KO GBM, collagen IV alignment was split, and irregular network formation (white arrows) was observed for collagen IV, collagen IV α3, α4, and α5 stainings, which showed disrupted network formation. Microscopy was performed with a LSM 800 with Airyscan using a ×63 objective, a digital zoom of ×8, and ×4 linear expansion of tissues. (**B**) Quantification of irregular network formation of collagen IV. Randomly chosen glomeruli from WT and KO mice were evaluated for linear and irregular collagen IV network formation. The graph shows that collagen IV network formation in KO GBM was significantly disrupted when compared with that in WT GBM, indicating that collagen IV α3, α4, and α5 proteins were unable to form a proper network in the absence of P3H2. *n =* 3. Graphs show the mean ± SD. ****P < 0.0001, by unpaired, 2-tailed Student’s *t* test.

We performed high-resolution imaging of WT and KO mice GBM using expansion microscopy to evaluate network formation of collagen IV α3α4α5. We stained WT and KO tissues for native collagen IV and collagen IV α3, α4, and α5 proteins, individually. Expansion of the tissue allowed for high-resolution evaluation of the collagen IV network. The results showed that in WT GBM, collagen IV α3, α4, and α5 proteins generated a straight collagen fiber network. However, straight fiber formation was disrupted in KO GBM. There was splitting and waving of collagen IV within the network of collagen IV α3α4α5 (Figure 4A). In WT glomeruli, we observed an abnormal GBM pattern in only 3.1% of the examined glomerular areas. However, in KO glomeruli, approximately 76% of all regions had GBM alterations. Together, these data clearly show that disruption of collagen IV network formation was more frequently observed in KO glomeruli than WT glomeruli, indicating a direct effect of P3H2 on collagen IV network formation (Figure 4B).

### Age-dependent progression of TBMN to FSGS is observed in P3h2^ΔPod^ mice.

**Figure 5:**
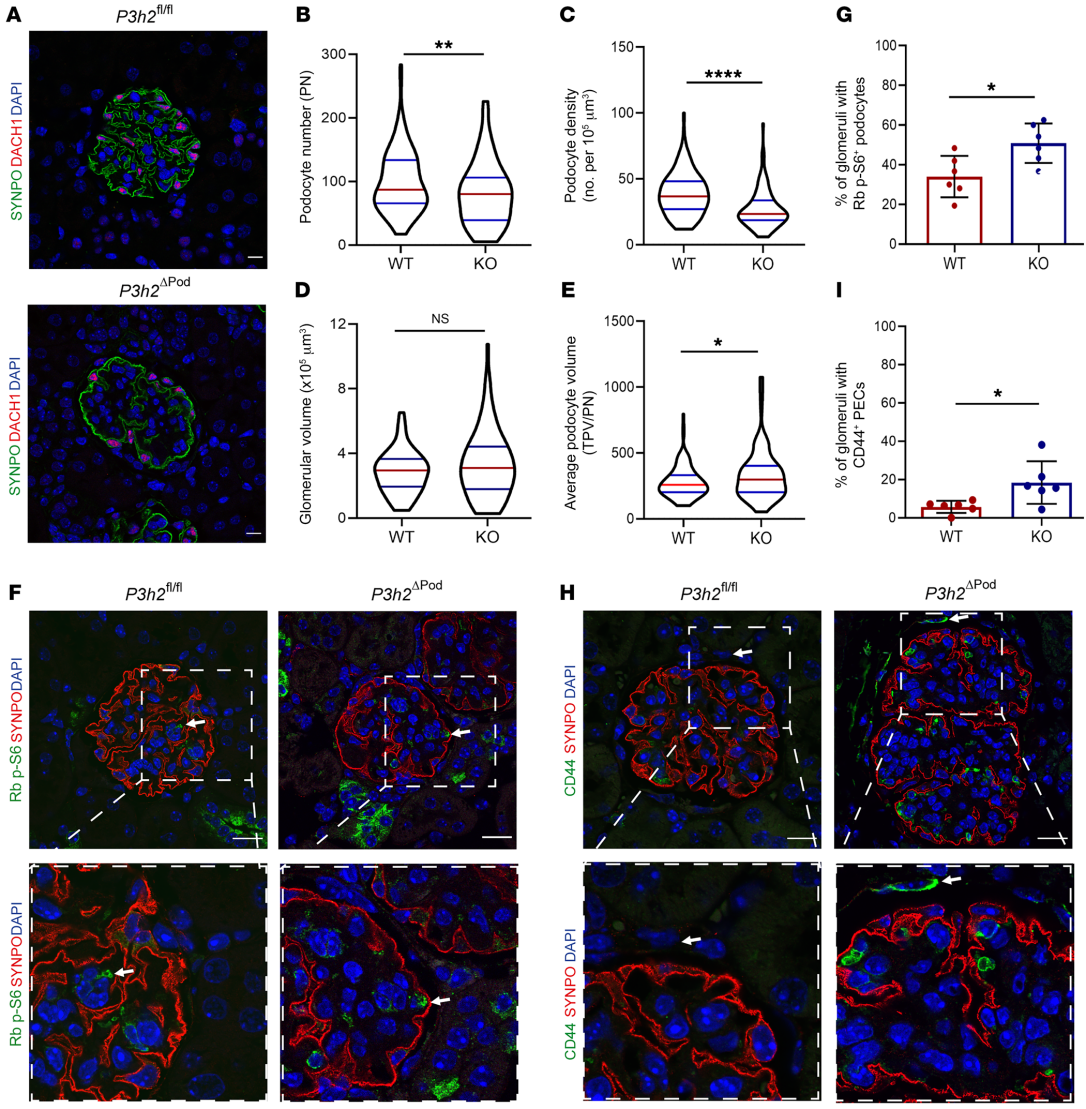
Podocyte morphometric analysis of *P3h2^ΔPod^* and *P3h2^fl/fl^* mice. (**A**) Representative immunofluorescence images of WT and KO mouse kidney tissue stained for SYNPO, DACH1, and DAPI. The images were used for the measurement of podocyte number, podocyte density, glomerular volume, and average podocyte volume (average podocyte volume = total podocyte volume/podocyte number [TPV/PN]). Scale bar: 10 μm. (**B**) Podocyte numbers for WT and KO mice. The number of podocytes was significantly decreased in KO mice glomeruli, indicating podocyte loss. (**C**) Podocyte density for WT and KO mice. In KO mouse glomeruli, the podocyte density was significantly decreased compared with that of WT glomeruli, again indicating podocyte loss. (**D**) Glomerular volume for WT and KO mice. There was no significant difference in glomerular volumes between WT and KO mice. (**E**) Average podocyte volume for WT and KO mice. A significant increase in the average podocyte volume was observed in KO mouse glomeruli, indicating podocyte hypertrophy. (**F**) Podocyte hypertrophy evaluation for *P3h2^ΔPod^* and *P3h2^fl/fl^* mice using immunofluorescence staining for ribosomal (Rb) p-S6, SYNPO, and DAPI. Representative immunofluorescence images show Rb p-S6 (green) in podocytes from both WT and KO mice (white arrows). Scale bars: 20 μm; inset zoom, ×5. (**G**) Quantification of immunofluorescence images showed a significant increase in podocyte hypertrophy in KO glomeruli when compared with WT glomeruli. (**H**) PEC activation analysis of *P3h2^ΔPod^* and *P3h2^fl/fl^* mice via immunofluorescence staining for CD44, SYNPO, and DAPI. Representative immunofluorescence images for KO mice show CD44 (green) signal in PECs (white arrow), indicating PEC activation. Representative immunofluorescence images for WT mice show no CD44^+^ signal in PECs (white arrow). Scale bars: 20 μm. Inset zoom, ×5. (**I**) Quantification of immunofluorescence images indicated a significant increase in the percentage of glomeruli with activated PECs in KO mice when compared with those of WT mice. *n =* 6. Data show the mean ± SD. *P < 0.05, by unpaired, 2-tailed Student’s *t* test. Graphs in **B**–**E** show the median ± IQR (*n* ≥6); *P < 0.05, **P < 0.01, and ****P < 0.0001, by Mann-Whitney *U* test. Graphs in **G** and **I** show the mean ± SD (*n =* 6).

We evaluated podocyte injury in 48-week-old *P3h2*^ΔPod^ mice using podocyte morphometric analysis (podometrics). We used podometrics to calculate podocyte numbers, podocyte density, glomerular volume, and the average podocyte volume. Immunofluorescence staining showed reduced podocyte numbers (Figure 5A). Podocyte numbers were significantly decreased in KO glomeruli when compared with WT glomeruli. The median values per KO glomerulus were 80 (*n =* 6) podocytes and 87 (*n =* 6) podocytes per WT glomerulus (*P <* 0.01; Figure 5B). The podocyte density was 23.3/μm^3^ (*n =* 6) in KO mice and 36.7/μm^3^ (*n =* 6) in WT mice (*P <* 0.0001; Figure 5C). Podocyte numbers and density were significantly decreased in KO glomeruli when compared with WT glomeruli. Glomerular volumes were not significantly changed between KO and WT glomeruli, within median values of 3.16 × 10^5^ μm^3^ (*n =* 6) for KO glomerular volumes and 2.95 × 10^5^ μm^3^ (*n =* 6) for WT glomerular volumes (*P >* 0.5; Figure 5D). Last, the average podocyte volume of the KO podocytes was 296.8 μm^3^ (*n =* 6) compared with 256 μm^3^ (*n =* 6) in WT podocytes (*P <* 0.05; Figure 5E). The significantly increased average podocyte volume in KO podocytes suggested compensatory podocyte hypertrophy. To confirm this finding, we performed phospho-ribosomal protein S6 staining as a marker of podocyte hypertrophy in KO and WT tissue and detected phospho-S6 (p-S6) protein at different levels between the genotypes (Figure 5F). Quantification of p-S6^+^ glomeruli revealed a significantly higher percentage (50.8% ± 9.9%) in KO mice than in WT mice (34.1% ± 10.4%) (*P <* 0.05; Figure 5G). Taken together, these data show that the KO mouse glomeruli lost podocytes and used podocyte hypertrophy as a compensatory mechanism before glomerular injury occurred.

We also observed a marked increase in the thickness of Bowman’s capsule in TEM micrographs. Activated parietal epithelial cells (PECs) secrete more extracellular matrix (ECM), causing this increase in thickness of Bowman’s capsule at the beginning of FSGS development (19). To confirm this finding, CD44, a PEC activation marker, was evaluated in KO and WT glomeruli using immunofluorescence staining. We detected activated PECs in KO glomeruli, while in WT glomeruli, they were in an inactive state (Figure 5H). The percentage of glomeruli with active PECs for KO mice was 18.4% ± 11.2% (*n =* 6) and 5.7% ± 3.2% (*n =* 6) for WT mice (*P <* 0.05; Figure 5I). This significant increase indicated a mild state of PEC activation, which correlated well with our PAS and TEM findings at 48 weeks.

### Differential quantitative GBM and ECM proteome analysis reveals that P3H2 modifies basement membrane composition.

**Figure 6:**
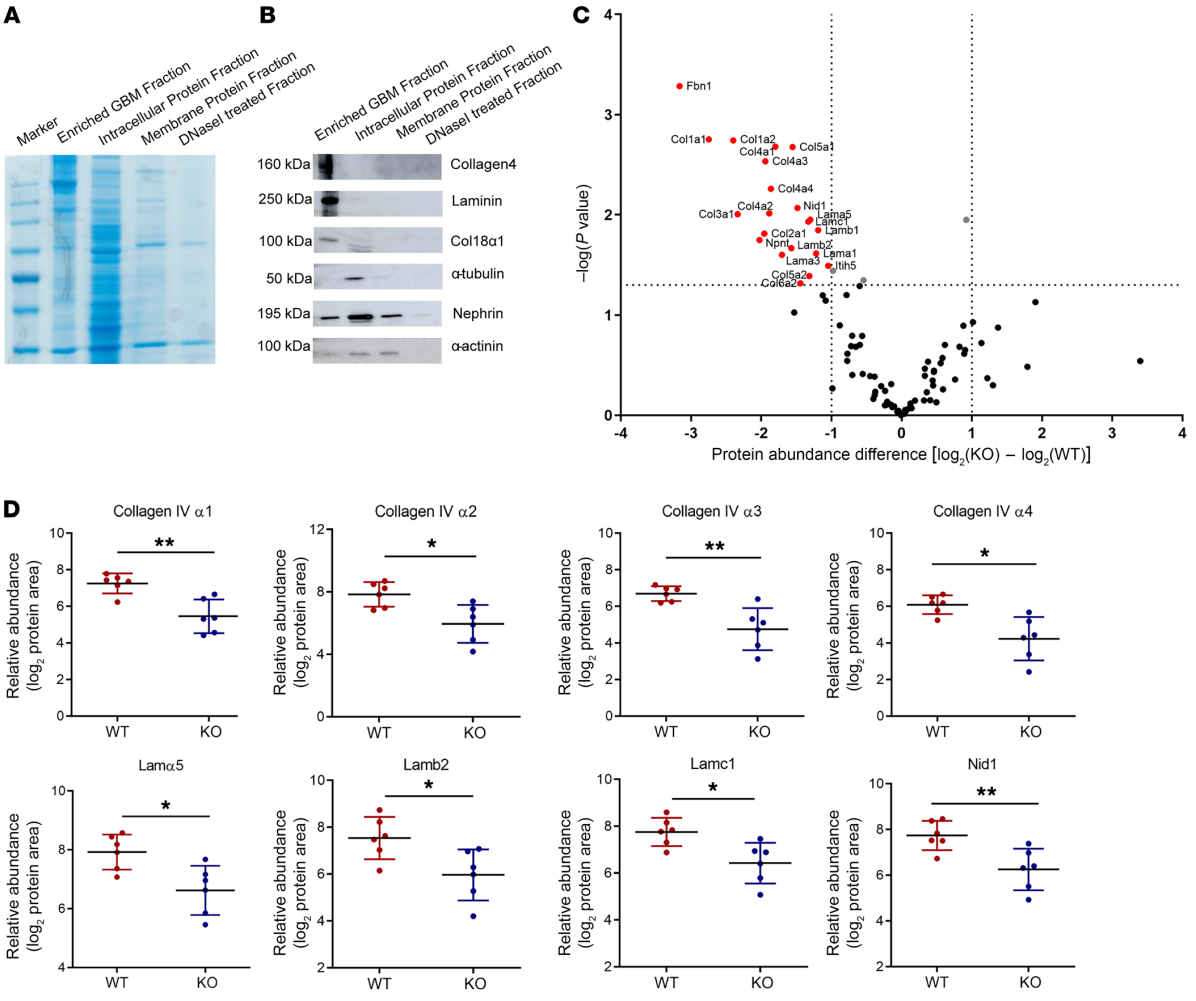
Relative quantitative proteomics of the GBM of *P3h2^ΔPod^* and *P3h2*^fl/fl^ mice. (**A**) Coomassie blue staining of enriched GBM and further fractions collected during GBM isolation. The GBM fraction had high-molecular-weight protein bands indicating that the isolated GBM was enriched with ECM proteins. The intracellular protein fraction had protein bands of variable size, showing that during isolation, many intracellular proteins were separated from the enriched GBM. (**B**) Western blot analysis of the GBM and other fractions for quality control of isolated GBM. ECM proteins were detected in high abundance in the enriched GBM fraction. Intracellular and transmembrane proteins were in low abundance or not detected in the enriched GBM fraction when compared with intracellular and membrane protein fractions. (**C**) Volcano plot of the relative quantitative GBM proteome. The *x* axis shows log_2_ fold changes in the abundance of WT and KO GBM proteins, and the *y* axis shows the *P* values for the GBM proteins. The abundance of the main structural GBM proteins was decreased in KO mice, as shown in the left portion of the plot. The vertical dotted line marks the –log(*P* value) cutoff of 1.3, above which all proteins are considered statistically significant; the dotted horizontal lines indicate the protein abundance difference [log_2_(KO) – log_2_(WT)] cutoff of less and –1 or greater than 1. Proteins with a –log(*P* value) of greater than 1.3 and a difference 1 or less are highlighted in red. (**D**) Dot plots of the structural proteins in the GBM showing a difference in their abundance. Comparison of the relative abundance values of the GBM proteome of each mouse group shows a significant decrease in the main GBM structural proteins. *n =* 6. Graphs show the mean ± SD. *P < 0.05 and **P < 0.01, by unpaired, 2-tailed Student’s *t* test.

We evaluated the protein abundance in the GBM using differential quantitative proteomics. As shown with Coomassie blue staining of an SDS gel, a high enrichment of large-sized GBM proteins could be achieved using our GBM isolation protocol. The cell’s lysate lanes had proteins at various sizes, indicating that many of the intracellular proteins were removed during isolation (Figure 6A). Western blot data showed that the enriched GBM had a high abundance of collagen IV and laminin, whereas intracellular protein (α-tubulin) was lacking when compared with whole-cell lysates, indicating a high quality of the isolated GBM (Figure 6B). *P3H2*-KO human immortalized cell lines were generated using CRISPR/Cas9 genome editing technology to evaluate ECM changes in vitro (Supplemental Figure 4A). We confirmed the lack of P3H2 protein in the generated cell lines (Supplemental Figure 4, B and C). Cellular assays were performed, which showed a similar cellular behavior of the generated KO clones before continuing with ECM analysis (Supplemental Figure 4, D–F). The same ECM quality indicators were detected in isolated ECM from the cell lines (Supplemental Figure 5A). The comparison of the relative abundance of GBM proteins in KO and WT mice and ECM proteins in cell lines is presented in a volcano plot in Figure 6C. The volcano plot of the GBM and ECM proteomes showed that the relative abundance of the main structural proteins significantly decreased in the GBM of KO mice and the ECM of KO cells (Figure 6C and Supplemental Figure 5B). Collagen IV α1 and α2 were downregulated in ECM of KO mice. The collagen IV subchains, laminin 521, and nidogen 1 were the most affected and decreased proteins in the GBM of KO mice. The relative abundance values are given in a dot plot analysis for each mouse group (Figure 6D). We hypothesize that the reduced abundance of these proteins could be a reason for the decreased GBM thickness observed in KO animals at 6 weeks and 28 weeks of age.

### 3Hyp of proline residues in collagen IV α2, α3, and α4 peptides is affected by P3h2 deletion.

**Figure 7:**
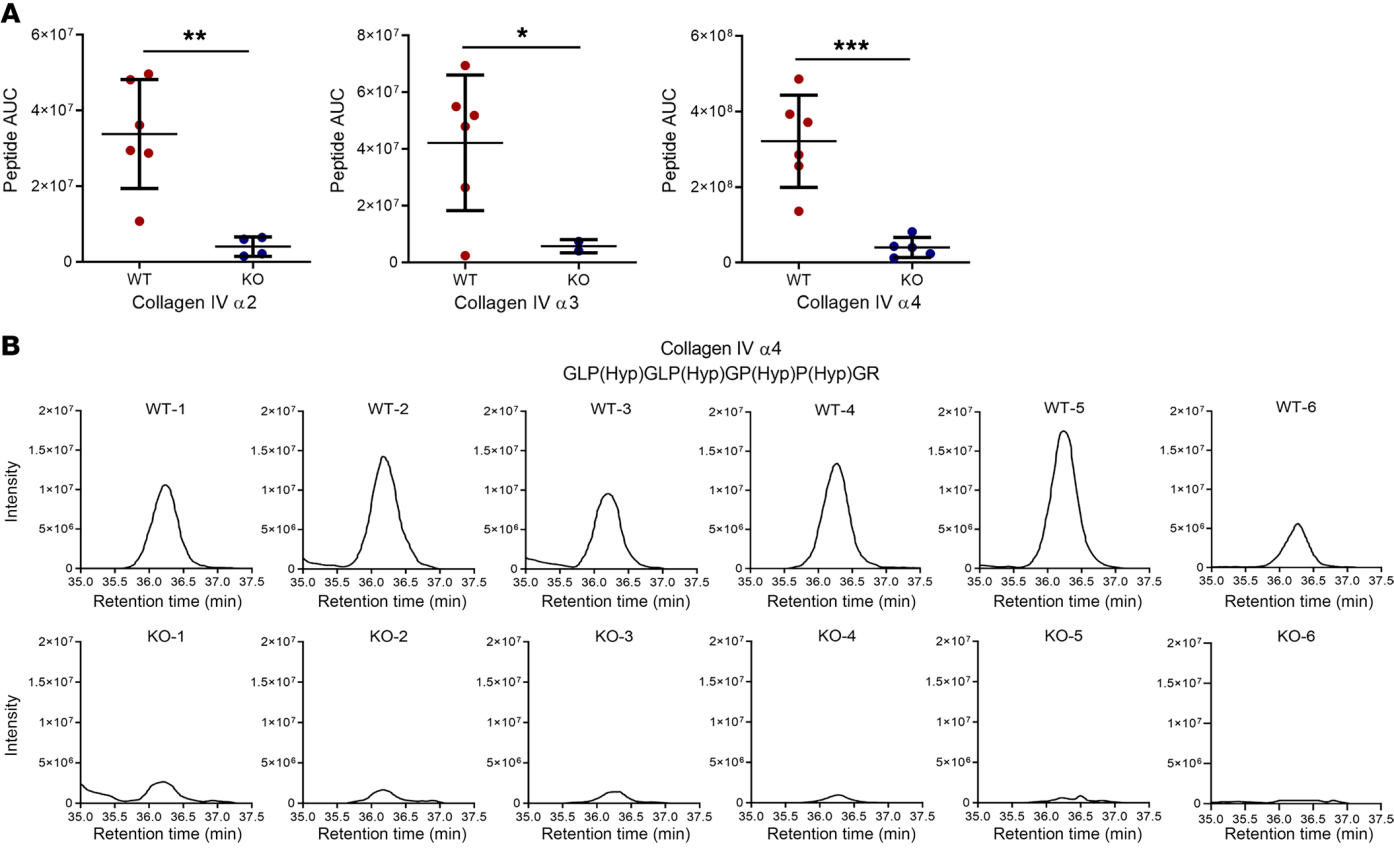
3Hyp analysis of collagen IV α2, α3, and α4. (**A**) Three different collagen IV peptides were chosen to show the effect of *P3h2* deletion on 3Hyp of proline residues. It shows that in KO GBM, selected collagen IV α2, α3 and α4 peptides had significantly less 3Hyp in proline residues than did WT GBM. (**B**) Individual 3Hyp intensity graphs of the collagen IV α4 peptide (GLP(Hyp)GLP(Hyp)GP(Hyp)P(Hyp)GR). 3Hyp were analyzed by measuring the intensities. In the KO collagen IV α4 peptide, the intensities were too low to detect when compared with WT, indicating an absence of 3Hyp on the proline residue. *n =* 6. Graphs show the mean ± SD. *P < 0.05, ** *P* < 0.01, and ***P < 0.001, by unpaired, 2-tailed Student’s *t* test.

P3H2 hydroxylates the 3′ of proline residues of collagen IV. Therefore, we aimed to evaluate the effect of an absence of P3H2 on the 3Hyp of proline residues of collagen IV. 3Hyp intensities were measured by mass spectrometry, which showed that the intensities of 3Hyp proline for collagen IV α2, α3, and α4 peptides were significantly decreased in KO GBM when compared with those in WT GBM (Figure 7A). 3Hyp intensities of the proline residue of the collagen IV α4-GLP(Hyp)GLP(Hyp)GP(Hyp)P(Hyp)GR peptide were detected not at all or only at a very low intensity for KO GBM when compared with WT GBM (Figure 7B). This indicates that P3H2 is the decisive enzyme for 3Hyp of proline residues of collagen IV.

### Rescue of the ECM phenotype of P3H2-KO podocyte cell lines via P3H2 overexpression.

**Figure 8:**
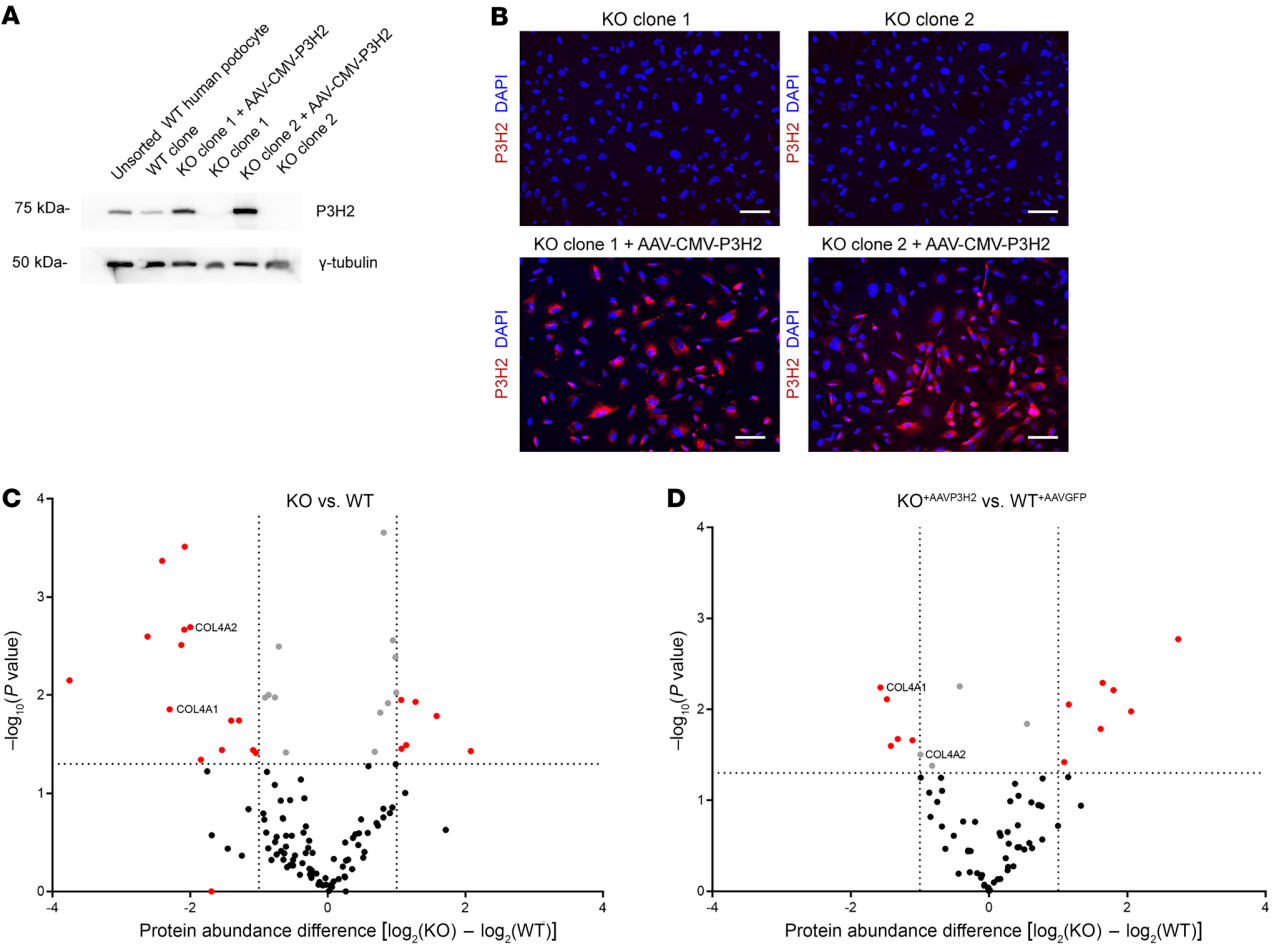
Rescue of the ECM phenotype of *P3H2*-KO podocyte lines via AAV-CMV-P3H2 infection. (**A**) *P3H2* expression was determined via Western blotting. P3H2 protein expression was detected in AAV-CMV-P3H2–infected KO cells. (**B**) Immunofluorescence staining of KO and infected cells. P3H2 localization was observed in infected cells with a 60%–70% infection efficiency. Scale bars: 20 μm. (**C**) ECM proteomics for WT and KO cells. The *x* axis shows log_2_ fold changes in the abundance of WT and KO GBM proteins, and the *y* axis shows the *P* values of the GBM proteins. In the volcano plot, downregulation of collagen IV α1 and α2 subchains in the KO ECM was observed when compared with WT. (**D**) ECM proteomics of AAV-CMV-GFP–infected WT and AAV-CMV-P3H2–infected KO cells. Volcano plot shows that collagen IV α2 was no longer significantly downregulate and that α1 was still significantly downregulated in the ECM of infected KO cells. However, the difference in abundances of collagen IV α1 and α2 subchains were decreased with infection of AAV-CMV-P3H2, indicating that *P3H2* reexpression increased the collagen IV α1 and α2 protein abundance and partially rescued the KO ECM phenotype. For **C** and **D**, the vertical dotted lines marks the –log(*P* value) cutoff of 1.3, above which all proteins were considered statistically significant; the dotted horizontal lines indicate the protein abundance difference [log_2_(KO) – log_2_(WT)] cutoff of less and –1 or greater than 1. Proteins with a –log(*P* value) of greater than 1.3 and a difference of 1 or less are highlighted in red.

To rescue the ECM phenotype of *P3H2*-KO podocyte cell lines, we generated an AAV-CMV-P3H2 vector. The KO cells were infected with AAV-CMV-P3H2, and, as a control, the WT cells were infected with AAV-CMV-GFP. We observed *P3H2* gene expression by Western blotting in the infected KO clones (Figure 8A). To determine infection efficiency, cells were stained with P3H2 and DAPI. Given that immortalized podocyte cell lines are difficult to transfect, we observed a reasonable infection efficiency of approximately 60%–70% (Figure 8B). We isolated ECM from infected cells to examine the effect of *P3H2* reexpression on ECM composition. We found that the protein abundance of collagen IV α1 and α2 subchains was downregulated in KO ECM compared with WT ECM (Figure 8C). The difference in abundance of collagen IV α1 and α2 between AAV-CMV-P3H2–infected KO cells and AAV-CMV-GFP–infected WT cells was decreased (Figure 8D). These data indicate that reexpression of the *P3H2* gene in KO cells could partially rescue the downregulation of collagen IV in the ECM.

## Discussion

Even though *P3H2* gene mutations were detected in patients with ocular phenotypes, the functional relevance of these mutations in the kidney remained unknown. High renal expression of this gene was shown, but further characterization was lacking (20). With the identification of a *P3H2* mutation in a cohort of patients with albuminuria, we investigated its importance for GBM and GFB homeostasis.

The expression profiles of glomerular cells showed that podocytes had the highest levels of *P3H2* expression. It is known that *COL4* gene mutations cause alterations in the GBM and that further progression disrupts glomerular integrity and leads to FSGS (21). In addition, disruption of the prolyl 4-hydroxylase gene in zebrafish led to a strong ocular phenotype, together with alterations of the pronephros glomeruli (22). Therefore, we generated *P3h2^ΔPod^* mice to characterize the effects of *P3h2* on GBM and GFB homeostasis as a collagen IV modifier in a mammalian system. With the findings at 6 weeks and 28 weeks, we could show that *P3h2^ΔPod^* mice had a TBMN-like phenotype, as a thinner GBM and microhematuria are hallmarks for this disease entity. At 48 weeks, we observed disease progression and a more severe phenotype in the KO mice when compared with earlier time points. An increase in urinary albumin and the presence of focal glomerulosclerosis were indicative of progressive damage to the glomerulus. Quantification of glomerulosclerosis showed significantly more sclerosis in the KO animals, even though there was still a high number of healthy glomeruli, which correlated well with the modest level of albuminuria being clearly below the nephrotic range. PECs line the Bowman’s capsule, and activation of PECs initiates glomerulosclerosis via the migration of these cells onto the glomerular tuft. PEC activation is a frequent finding in other kidney diseases, since the formation of sclerosis is a redundant pathophysiological trait. Even though some steps of this process are known today, several essential parts remain poorly understood (23). We could detect activated PECs in the glomeruli of *P3h2^ΔPod^* mice at 48 weeks, suggesting activation of this common sclerosis mechanism in these mice.

Glomerular integrity is regulated through many signaling pathways, and mammalian target of rapamycin complex 1 (mTORC1) signaling is one of them. This pathway regulates podocyte survival and growth. Its up- and downregulation can induce podocyte stress. Overactivity of mTORC1 signaling in podocytes has been observed in many kidney diseases and is thought of as a common hypertrophic stress pathway of podocytes (24). We found podocyte loss and podocyte hypertrophy in the *P3h2^ΔPod^* mice glomeruli at 48 weeks.

Differential quantitative GBM proteome analysis showed that collagen IV subchains and other structural GBM proteins were significantly decreased in the KO mice (Figure 6D and Figure 7A). Moreover, collagen IV α1 and α2 abundance was decreased in the ECM of *P3H2*-KO immortalized podocyte cell lines. This finding might explain the thinner GBM observed in the KO mice. We observed a decrease not only in collagen IV, but also in collagen IV interactors (i.e., collagen XVIII α1 and nidogen 1). These findings might support a functional role of 3Hyp in protein-protein interaction. Indeed, a detailed analysis of collagen IV network formation using high-resolution expansion microscopy showed that the formation was unaffected in WT mice but highly disrupted in KO mice.

There are 2 explanations for the decreased abundance of collagen IV subchains in both the ECM and the GBM. First, there might be less secretion of collagen IV from podocytes to the extracellular space, since a lack of 3Hyp might affect its proper secretion. Second, collagen IV lacking 3Hyp within the GBM might have a shorter half-life than properly hydroxylated collagen IV. Lack of 3Hyp might make collagen IV less stable or might prevent proper cross-linking, which could induce its degradation by MMPs. MMPs were not increased in their abundance according to our proteomics data, but their activity could be increased. However, more detailed investigations are necessary to support these hypotheses.

Our index patient had FSGS, and progression of the disease resulted in end-stage kidney disease. The subsequently identified patients had a milder phenotype. There are several reasons to explain the differences between these patients. First, our index patient and the subsequently identified other families are from different ethnic populations, and their genetic background is different. Second, it is known that environmental factors have an influence on disease mechanisms and can affect its progression. Last, the identified *P3H2* mutation was different in our index patient compared with that in the other 2 families. Different mutations interfere with protein structure and affect the interaction, function, and activity of the mutated protein (25). These genetic reasons, together with unknown environmental effects, might explain the differences in disease severity between our index patient and the subsequently identified patients. To take into account the long latency of overt disease appearance in our mouse model, we propose a 2-hit hypothesis, in which genetic predisposition and additional environmental, genetic, and immunological factors may interact to induce overt disease.

Our findings underline the importance of P3H2 as a GBM modifier. an absence of P3H2 induces TBMN, and over time this phenotype follows a common pathophysiological process leading to the development of FSGS. The comprehensive data on *P3h2^ΔPod^* mice and the identified patient families support the functional relevance of P3H2. We show that a GBM modifier can disrupt GFB homeostasis and induce a kidney phenotype. Further investigations will be necessary to better understand the molecular mechanisms driving this TBMN and FSGS phenotype.

## Methods

Additional details can be found in the Supplemental Methods.

### Generation of P3h2^ΔPod^ mice.

Mice were housed in a specific pathogen–free (SPF) facility with free access to chow and water and a 12-hour day/12-hour night cycle. Breeding and genotyping were done according to standard procedures. A conditional *P3h2*-KO mouse was generated from embryonic stem (ES) cells from the UC Davis KOMP (Knock-Out Mouse Project) repository [P3h2^tm1aWtsi^ (MGI: 4364924)]. B6.Cg-Tg(Pgk1-FLPo)10Sykr/J mice, obtained by the Central University Hospital Hamburg Eppendorf mouse facility from The Jackson Laboratory, were used to generate a conditional allele of *P3h2*. Floxed allele *P3h2* (*P3h2^fl/fl^*) mice were bred with *Nphs2*^Cre+/+^ (TghNphs2-Cre295^Lbh^) mice (a gift from M.J. Moeller, RWTH University, Aachen, Germany) to generate podocyte-specific *P3h2–*KO (*P3h2^ΔPod^*) mice.

### Identification of a patient with the P3H2 mutation.

An expression-based candidate gene approach was applied to identify novel genes in a cohort of patients with albuminuria. A corresponding next-generation sequencing–based (NGS-based) panel for the parallel analysis of all previously described and potentially new causative genes was established. Candidate genes were selected using a comparative and sequential algorithm from genes heavily expressed in mouse podocytes. With the help of publicly accessible databases, the 750 most enriched transcripts in mice were tested for (a) their expression in human kidney cortex; (b) their expression in human glomerulus; (c) their occurrence in the murine podocyte proteome; (d) a significant regulation in human proteinuric diseases; or (e) a statistical overrepresentation in the respective GO term–based gene clusters. The most promising candidates were chosen according to their scores. The NGS technologies and comprehensive bioinformatics analyses utilized in this project are described in detail elsewhere (26, 27). Currently, our custom-designed panel contains more than 600 genes described and associated with kidney disease or allied disorders as well as the corresponding flanking intronic sequences. The panel design is constantly updated by surveillance of the current literature as well as enriched by targets in noncoding regions for the described variants listed in well-accepted databases like the Human Gene Mutation Database (HGMD)and ClinVar. Moreover, the design is optimized in low-performance regions as well as in critical regions. DNA samples were pooled and sequenced in a multiplexing procedure. DNAs were enriched using a sequence capture approach and sequenced using Illumina sequencing-by-synthesis technology with an average coverage of more than 300X for a targeted panel setup. Raw data were processed according to bioinformatics best practice procedures. Mapping and coverage statistics were generated from the mapping output files using standard bioinformatics tools (e.g., Picard). High and reproducible coverage achieved by our sequencing approach enabled copy number variation (CNV) analysis.

Performance of the wet-lab and bioinformatics processes was validated and controlled according to national and international guidelines reaching high sensitivity for single nucleotide variants (SNVs), indels, and CNVs using well-established reference samples as well as a large cohort of positive controls, especially for CNVs. For interpretation of the identified variants, we have developed our own published bioinformatic algorithms using a stepwise filtering process conducted by an experienced team of scientists and supported by various bioinformatics decision tools. Sequence variants of interest were verified by Sanger sequencing if the NGS results failed internal validation guidelines. If other family members were available, segregation of sequence variants with the disease was further assessed.

The *P3H2* gene had one of the highest GO term values in the analysis. A young female patient with SRNS and histologically diagnosed with FSGS along with early cataract development and high myopia presented with a homozygous *P3H2* mutation and did not harbor any other pathogenic variant in known causative FSGS and SRNS genes.

### Statistics.

The data in the diagrams of the results section are shown in different types of plots. All statistical analysis were performed and plots were prepared using GraphPad Prism, version 8.4.0 (GraphPad Software). The data are presented as the mean with SD or the median with the IQR. An unpaired, 2-tailed Student’s *t* test and a Mann-Whitney *U* test were used to test for significance between the experimental and control groups. When 3 or more groups were assessed, 1-way ANOVA with Tukey’s multiple-comparison post hoc test was used. A *P* value of less than 0.05 was set as the significance level. A *P* value of less than 0.01 was considered very significant, and *P* values of less than 0.001 and less than 0.0001 were considered highly significant.

### Study approval.

All animal experiments were conducted according to the NIH’s *Guide for the Care and Use of Laboratory Animals* (National Academies Press, 2011), as well as the German law for the welfare of animals. Animal procedures were approved by the Regierungspräsidium Freiburg (G16-122) and BGV Hansestadt Hamburg (Ü 004/2018). All human samples in this study were obtained with written informed consent accompanying the patients’ samples. All clinical investigations were conducted according to the principles expressed in the Declaration of Helsinki and were approved by the IRBs of the State Key Laboratory of Medical Genetics (SKLMG) in Changsha, Hunan, China, and the King Faisal Specialist Hospital and Research Center in Riyadh, Saudi Arabia (15, 18).

## Author contributions

All authors read and revised the manuscript before submission. HA, CK, S Lu, S Liu, DK, OK, GW, JMD, MM, TW, and FG performed experiments. OK performed all TEM observations and analysis. MM, CK, and HS performed differential quantitative mass spectrometry–based proteome analysis, data interpretation, and statistical analysis of the proteomics data and provided expertise. DK and VGP assisted with the expansion microscopy, morphometrics, and immunofluorescence analyses. S Lu and S Liu helped with the FACS analysis. KA and KB provided the patient biopsy tissue. MG, TW, and CB performed genetic analysis for the detection of novel genes. PT, ZMH, SMA, and AOK provided *P3H2* human patient and urine data. HA, CB, TBH, and FG were responsible for conceptualization, interpretation of data, writing and editing the manuscript. TBH and FG supervised the study.

## Supplementary Material

Supplemental data

## Version 1 05/02/2022

Electronic publication
