# Supplementary material for: Loss of the collagen IV modifier prolyl 3-hydroxylase 2 causes thin basement membrane nephropathy
Source: J Clin Invest. 2022 May 2;132(9):e147253. doi: 10.1172/JCI147253 (PMC9057608; doi:10.1172/JCI147253)
Supplement: Supplemental data [file jci-132-147253-s230.pdf]

# Loss of the collagen IV modifier Prolyl 3-Hydroxylase 2 (P3H2) causes thin basement membrane nephropathy

*Hande Aypek[1], Christoph Krisp[2], Shun Lu[1], Shuya Liu[1], Dominik Kylies[1], Oliver Kretz[1], Guochao Wu[1], Manuela Moritz[2], Kerstin Amann[3], Kerstin Benz[4], Ping Tong[5], Zheng-mao Hu[6], Sulaiman M. Alsulaiman[7], Arif O. Khan[8,9], Maik Grohmann[10], Timo Wagner[10], Janina Müller-Deile[11], Hartmut Schlüter[2], Victor G. Puelles[1], Carsten Bergmann [10,12], Tobias B. Huber[1] and Florian Grahammer[1]*

[1]III. Department of Medicine, University Medical Center Hamburg-Eppendorf, Hamburg, Germany.

[2]Institute of Clinical Chemistry and Laboratory Medicine, Mass Spectrometric Proteomics Group, University Medical Center Hamburg-Eppendorf, Hamburg, Germany.

[3]Dept. of Nephropathology, Institute of Pathology, University of Erlangen, Erlangen, Germany.

[4]Department of Pediatrics, University of Erlangen, Erlangen, Germany.

[5]Department of Ophthalmology, the Second Xiangya Hospital, Central South University, Changsha, Hunan, China

[6]Center for Medical Genetics, School of Life Sciences, Central South University, Changsha, Hunan, China.

[7]Vitreoretinal Division, King Khaled Eye Specialist Hospital, Riyadh, Saudi Arabia

[8]Eye Institute, Cleveland Clinic Abu Dhabi, Abu Dhabi, United Arab Emirates

[9]Department of Ophthalmology, Cleveland Clinic Lerner College of Medicine of Case Western University, Cleveland, Ohio, USA

[10]Medizinische Genetik Mainz, Limbach Genetics, Mainz, Germany.

[11]Department of Nephrology, Friedrich-Alexander-Universität Erlangen-Nürnberg, Erlangen, Germany

[12]Department of Medicine, Division of Nephrology, University Hospital Freiburg, Freiburg, Germany.

## Supplemental Data

**Supplement Figure 1: Patients who have ocular abnormalities related to a *P3H2* mutation.**

| **Sex** | **Age** | ***P3H2* Mutation** | **Microalbuminuria/ Microhematuria** |
|---|---|---|---|
| Family 1 (Guo H et al, 2014*, Clinical Genetics)* | | | |
| Male | 67 | c.13C>T; p.Q5X | ++ |
| Female | 51 | c.13C>T; p.Q5X | +++ |
| Family 2 | | | |
| Female | 21 | c.679G>T; p.Glu277* | + |
| Female | 17 | c.679G>T; p.Glu277* | - |
| Female | 13 | c.679G>T; p.Glu277* | + |
| Female | 11 | c.679G>T; p.Glu277* | + |

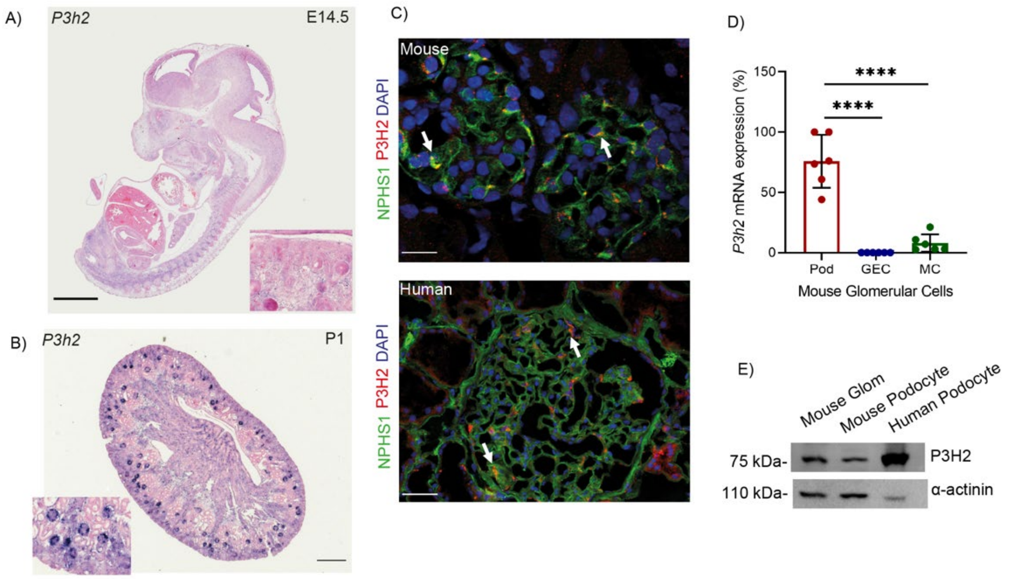


**Supplement Figure 2: *P3h2* expression in kidney**. A) ISH of E.14 mouse embryo for *P3h2. P3h2* RNA localization couldn't be detected in kidney at E14.5. Scale bar, 2 mm. B) ISH of P1 mice kidney for *P3h2*. *P3h2* RNA was localized in the outer cellular layer of glomeruli. Scale bar, 500 µm. C) Immunofluorescence staining of human and mice paraffin-embedded kidney tissue with P3H2, NPHS1, and DAPI. White arrows indicate the co-localization of P3H2 and NPHS1 in both species. Scale bar, 20 µm. D) *P3h2* mRNA expression in sorted mouse glomerular cells determined by qPCR. Pods have the highest expression level of *P3h2*. Graph shows mean ± SD, n=6, one-way ANOVA with Tukey multiple comparison post hoc test, p-value ****< 0.0001. E) P3H2 protein expression is detected in mouse glomeruli, immortalized mouse and human podocyte cell lines using Western blot.

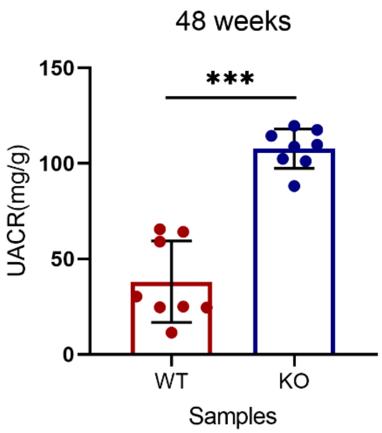


**Supplement Figure 3: UACR analysis of WT and KO mice at 48w.** Albuminuria was observed with an ACR of 107.7 ± 10.3 mg/g for KO and 38.1 ± 21.3 mg/g for WT mice at 48w. Graph shows mean ± SD, n=8 3, unpaired 2-tailed t-test, p-value ***< 0.001.

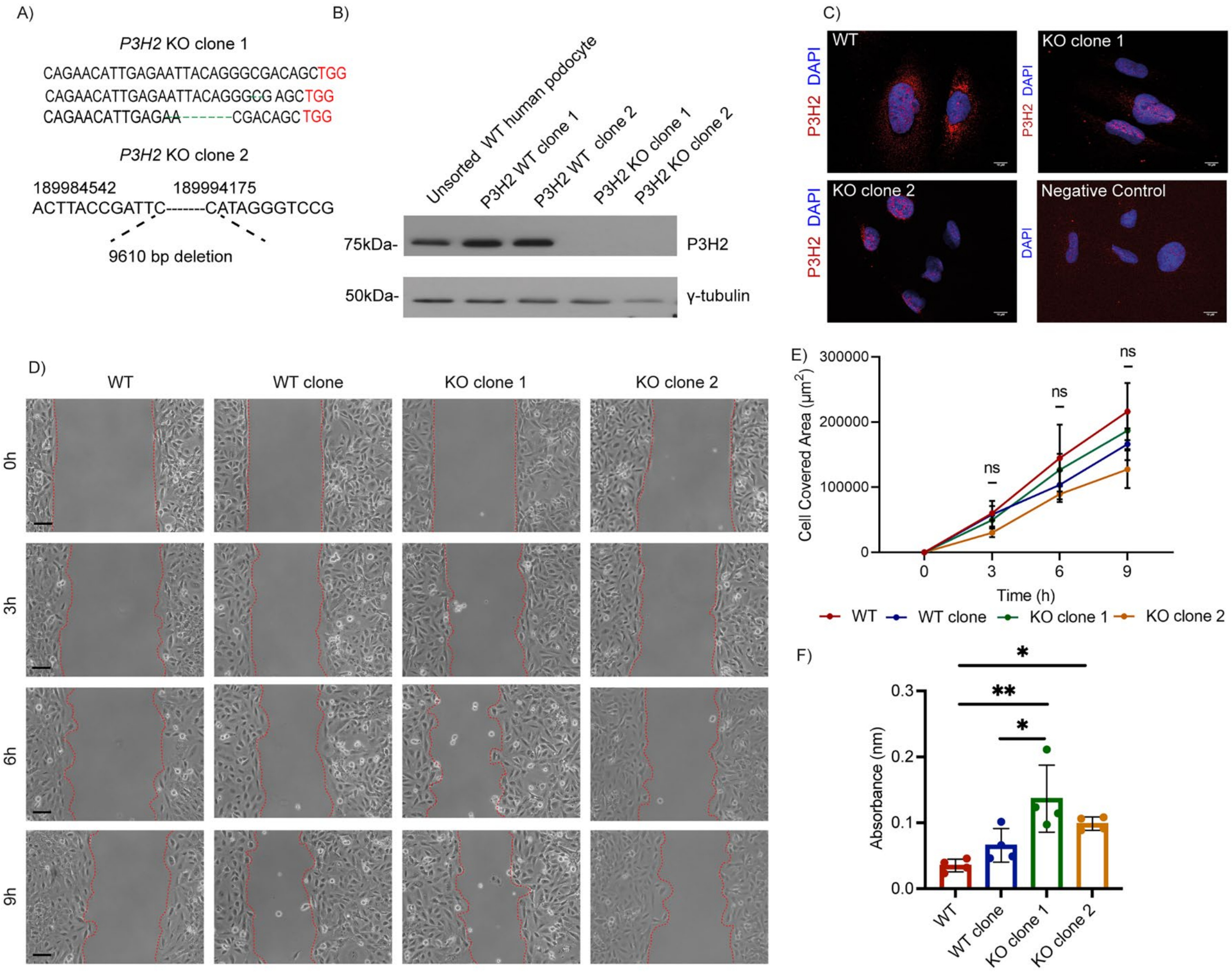


**Supplement Figure 4: Generation and characterization of *P3H2* KO immortalized human podocyte cell lines**. A) Edited P3H2 gene sequences via Cas9 nuclease. There were a 2 bp and a 5 bp deletion on the alleles of KO clone 1. There was a 9610 bp deletion on the alleles of KO clone 2. B) Protein level confirmation of P3H2 absence in KO clones. Western blot analysis of WT and KO clones for P3H2 protein expression. There was no protein expression in KO clones when compared to WT and WT clone. C) Immunofluorescence staining of WT and KO clones with P3H2 and DAPI. Endoplasmic reticulum-like localization of P3H2 was lacking in KO clones when compared with WT. Scale bar 10 µm. D) Migration assay of *P3H2* WT and KO podocyte cell lines. Representative images of the cell migration at 0h, 3h, 6h, and 9h. Red dashes show the cell borders. Scale bar 50 µm E) Quantification of the migrated area. There was no significant difference between KO clones and WT and WT clone regarding cell covered area during 9h migration. F) Adhesion assay of the *P3H2* KO and WT podocyte cell lines. KO clones had higher adhesion capacity than unsorted WT and WT clone. Graphs show mean ± SD, n=4, one-way ANOVA with Tukey multiple comparison post hoc test, p-value ns > 0.05, * < 0.05, ** <0.01.

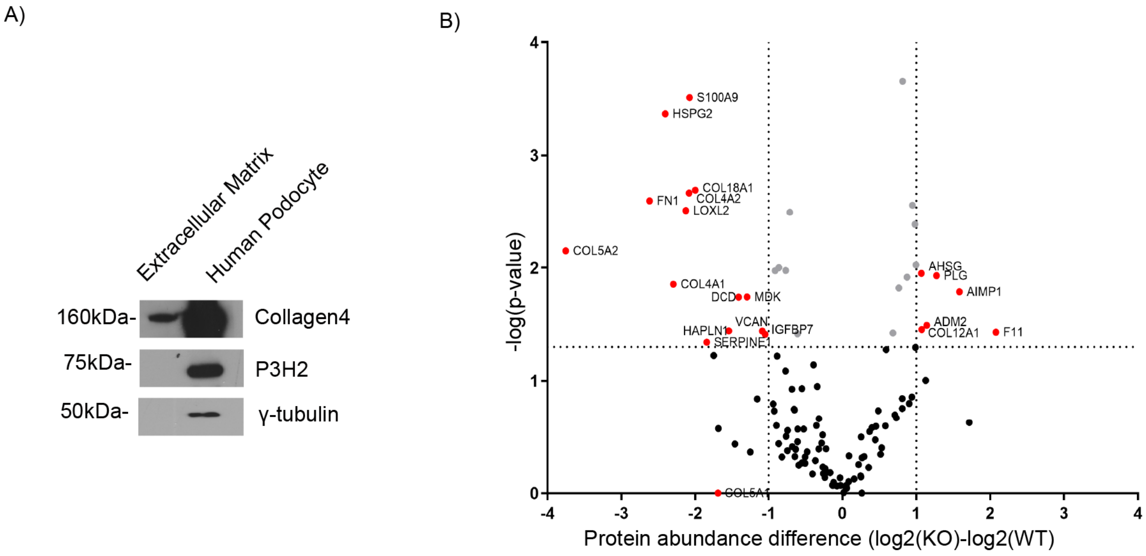


**Supplement Figure 5: Comparison of the relative abundance of the ECM proteome of *P3H2* KO immortalized human podocyte cell lines with WT immortalized human podocyte cell lines**. A) Western blot of isolated ECM for quality control. Collagen4 as an ECM protein was detected in the ECM. Intracellular proteins were not detected in ECM when compared with intracellular proteins (P3H2 and gamma-tubulin) of human podocyte lysate. B) Volcano plot of the relative quantitative composition of the ECM proteome of *P3H2* KO cells compared to WT cells. The x-axis represents the log2 fold change difference in abundance of proteins of WT and KO ECM. The y-axis represents the p-values of the proteins. The volcano plot shows downregulation of collagen IV $\alpha$1, collagen IV $\alpha$2 and collagen XVIII $\alpha$1 in KO ECM.

## Materials and Methods

### Animal experimentation

### Urine collection and urinalysis

To collect mouse urine, animals were held over a Petri dish and slight pressure was exerted manually on the bladder. The urine collected in the Petri dish was picked up with a pipette and transferred into a 1.5 ml Eppendorf tube. The urine was then stored at -20 °C until further evaluation or analyzed freshly after centrifugation to evaluate for red blood cells.

A homemade albumin ELISA was used for albumin measurement in the urine of mice at 6w, 28w and 48w. In short, a 96 well plate was coated with goat anti-mouse albumin antibody (Bethyl, Cat# A90-134A) in coating buffer (0.05M Carbonate-bicarbonate pH 9.6) as 1:100 dilution overnight at 4 °C. The following day, the coated plate was incubated with a post coat solution (50mM TBS, 1%BSA pH 8.0) for 30 min. Afterward, the plate was incubated with diluted urine (1:300) for 1h. After washing, the plate was incubated with the secondary antibody, goat anti-mouse Albumin-HRP, (Bethyl, Cat# A90-134P) for 1h. TMB substrate (Biomol, Cat# E102) was incubated for 3 min and the reaction was stopped by stop solution (2N $H_2SO_2$). The absorbance was measured at 450 nm in an ELx808 Absorbance Microplate Reader from Biotek Instruments.

A creatinine measurement kit (Labor und Technik, Cat# LT-CR 0106) was used according to the manufacturer's protocol to measure creatinine in mice urine at 6w, 28w and 48w. The measurement was taken at 562 nm in a TECAN Sunrise Basic microplate reader.

A urea measurement kit (Labor und Technik, Cat# LT-UR 0100) was used according to the manufacturer's protocol to measure urea in mice serum at 28w and 48w. The measurement was taken at 365 nm in a Thermo Fisher NanoDrop spectrophotometer.

A cystatin C measurement kit (Abcam, Cat# ab201280) was used according to the manufacturer's protocol to measure cystatin C in mice serum at 28w and 48w. The measurement was taken at 450 nm in a TECAN Sunrise Basic microplate reader.

Mouse urine at 48w was evaluated under a light microscope to count red blood cells at 40x magnification for hematuria. Dipsticks were used for qualitative measurement (Siemens Multistix 10SG, Cat# 01526748).

**Glomeruli Isolation and FACS**

Mice kidneys were perfused at 37°C with a magnetic nanoparticle solution (Dynabeads® M-450 Tosylactivated and M-450 Epoxy) via the renal arteries. The mouse cortex was digested with collagenase V (Col V) (5.5 ml HBSS+5.5 ml DMEM/F12+27.5 mg BSA+11 mg Col V). Tissue was homogenized by using a gentleMACS dissociator. Homogenized tissues were passed through a 100 µm cell strainer and centrifuged for 5 min at 4° C at 300 g. Glomeruli were collected with a DynaMag -2 Magnet and subsequently digested. The isolated glomeruli were resuspended in Collagenase II solution (300 U/ml Col II (Worthington Biochemical, Cat# LS004176), 5 U/ml Pronase E (Sigma-Aldrich, Cat# P6911), 1.25 U/ml Dispase II (Sigma-Aldrich, Cat# D4693), 50 U/ml DNase I (Roche, Cat#04716728001) in HBSS) for 40 min at 37 °C on a thermomixer shaker (1400 rpm). During incubation, different mechanical stresses were applied to detach cells. Isolated glomeruli were mixed by pipetting at 5, 10, 20, 25, and 35 min and sheered with a 27G

needle attached to a 1 ml syringe at 15 min of incubation. Also, cells were loosened at 10, 20, 30 min by vortexing. Digested glomeruli were pipetted up and down with a 200 µl pipette attached to a 1000 µl pipette at 30 and 40 min. After digestion, single cells were sieved through a 40 µm cell strainer and the cell suspension was centrifuged at 400 x g for 4 min at 4 °C. The cells were resuspended in staining buffer (BD, Cat# 554657) and beads were removed via a DynaMag -2 Magnet. Supernatant including all glomerular cells was taken and passed through a 35 µm cell strainer on a FACS tube and stained with PODXL-AF647, CD45-BV650 and CD105-PE antibodies for 30 min at 4 °C in the dark. After staining, cells were washed with staining buffer and centrifuged at 400 x g for 4 min at 4 °C. The cell pellet was resuspended in staining buffer and DAPI (0.08 ul/ml) was added to the cells 5 min before sorting. Immune cells and dead cells were excluded by CD45 and DAPI, respectively. 30.000 podocytes (PODXL+/CD105-), endothelial (PODXL+/CD105+) and mesangial cells (PODXL-/CD105+) were sorted in RNA lysis buffer via a BD AriaFusion Sorting device for subsequent RNA isolation.

**In situ hybridization**

ISH was performed for mouse embryo (E14.5) and P1 kidney. Probe design was performed by cloning *P3h2* specific primers into the pBSK II KS vector. Linearization and DIG RNA labeling was performed to finish probe synthesis. 10 µm tissue sections were immersed in 1x PBS for 5 min, 50 µl proteinase K (Roche, Cat# 3115887001) in 1x PBS for 5 min, 2 mg/ml glycine in PBS for 5 min, 4% PFA for 15 min, 0.25% acetic anhydride (Sigma Aldrich, Cat# A6404) for 10 min after deparaffinization. The slides were transferred into a hybridization buffer containing probes and incubated at 68 °C for 24 h. Afterward, the sections were incubated in TBST (Sigma Aldrich, Cat# T9039) three times

for 15 min each, blocking reagent for 30 min, Anti-DIG antibody solution for 2h, TBST four times 15 min each, NTMT for 15 min three times and BM purple (Roche, Cat# 1442074). After the color reaction, the sections were dehydrated and covered with Entellan mounting medium (Sigma Aldrich, Cat# 1079600500). The slides were analyzed using a Zeiss Axio Scope A1 microscope.

**Generation of *P3H2* KO immortalized human podocyte cell lines**

*P3H2* gene-specific gRNAs were designed and cloned into the Cas9 nuclease vector (Thermo Fisher Scientific, Cat# A21174). The conditionally immortalized human podocytes were kindly provided by M. Saleem (University of Bristol, UK). The cell line was transfected with the gRNAs cloned into the vector encoding Cas9. After quality control of the gRNAs via restriction assays, transfected cells were sorted in a 96-well plate in a 1 cell per 1 well manner. Surviving clones were screened for both genomic and protein level KO proof by Sanger sequencing and Western blot, respectively.

**Protein Isolation and Western blot**

The cells and glomeruli were lysed in RIPA solution (50mM Tris/HCl pH 7.5, 1mM EGTA, 1mM EDTA, 1% (w/v) Triton X-100, 0.1% SDS, 150 mM NaCl and 50mM NaF) with 0.1% 2-mercaptoethanol, 1mM sodium orthovanadate, protease inhibitor cocktail (Roche, Cat# 5892970001). For glomeruli, this mixture was homogenized with tissue grinders. After centrifugation, the supernatant was collected. 40 µg total protein lysates were loaded on SDS gels. After separation, the Trans-Blot® Turbo Transfer System (Bio-Rad, Cat# 1704150) was used to blot the proteins at 20V, 1.3 mA for 10 min. The membrane was blocked with 5% BSA for 1h at RT. The primary and secondary antibodies were incubated

overnight at 4° C and 45 min at RT, respectively. The protein bands were visualized via ECL on an Amersham Imager 600, GE Healthcare Life Sciences.

**Antibodies**

| Name of the antibody | Company, Catalog Number |
|---|---|
| **Primary antibodies** | |
| guinea pig anti-Nephrin | Progen, GP-N2 |
| rabbit anti-P3H2 | ProteinTech, 15723-1-AP |
| mouse anti-gamma-tubulin | Sigma-Aldrich, T5326 |
| rabbit anti-collagen 4 | Abcam, ab6586 |
| rabbit anti-Col18$\alpha$1 | Sigma-Aldrich, HPA011025 |
| mouse anti-alpha Actinin | Santa Cruz, sc-166524 |
| rabbit anti-Laminin | Abcam, ab11575 |
| mouse anti-alpha tubulin | Sigma-Aldrich, T9026 |
| guinea pig anti-Synaptopodin | SYSY, 163004 |
| rabbit anti-DACH1 | Sigma Aldrich, HPA012672 |
| rabbit anti- phospho-S6 Ribosomal Protein | Cell signaling, 2211 |
| rat anti-human collagen 4 alpha 3, H31 | Chondrex,7076 |
| rat anti-human collagen 4 alpha 4, H43 | Chondrex,7073 |
| rat anti-human collagen 4 alpha 5, H53 | Chondrex,7078 |

| Name of the antibody | Company, Catalog number |
|---|---|
| **Secondary antibodies** | |
| polyclonal goat Anti-rabbit IgG, HRP-linked Antibody | CST, 7074S |
| polyclonal Rabbit Anti-Guinea Pig Immunoglobulins/HRP | Dako, P0141 |
| polyclonal Goat Anti-Mouse Immunoglobulins/HRP | Dako, P0447 |
| mouse anti-goat IgG-HRP | Santa Cruz, sc-2354 |
| Alexa Fluor 488 Phalloidin | Thermo Fisher Scientific, A12379 |
| Alexa Fluor 647 anti-mouse/human CD44 | BioLegend, 103018 |
| Alexa Fluor 555 donkey anti-rabbit IgG | Thermo Fisher Scientific, A31572 |
| Alexa Fluor 488 goat anti-guinea pig IgG | Thermo Fisher Scientific, A11073 |
| Alexa Fluor 488 donkey anti-mouse IgG | Thermo Fisher Scientific, A21202 |
| PE anti-mouse CD105 Antibody | BioLegend, 120407 |
| Alexa Fluor 647 anti-mouse Podocalyxin Antibody | R&D, FAB1556R |
| Brilliant Violent 650 anti-mouse CD45 Antibody | BioLegend, 103151 |
| Alexa Fluor 555 goat anti-rat IgG | Thermo Fisher Scientific, A21434 |

**RNA isolation and qPCR**

RNeasy Micro Kit (Qiagen, Cat# 74004), ProtoScript® II First Strand cDNA Synthesis Kit (NEB, E6560S) and TaqMan Gene Expression Assays were used for RNA isolation, cDNA synthesis and qPCR analysis according to the manufacturer's protocol, respectively. P3h2 (Mm01342192_m1) and Gapdh (Mm99999915_g1) were the primers used in qPCR.

**Immunohistochemistry**

After deparaffinization and rehydration of tissue sections, the slides were immersed in 1% periodic acid solution for 15 min at RT and stained with Schiff's reagent for 45 min at RT. For counterstain, tissues were stained with haematin for 3 min at RT. The slides were immersed in 50% ethanol, 70% ethanol, 95% ethanol, 100% ethanol, and covered with Eukitt to fix the staining. Images were taken at the Zeiss Axio Scope A1 microscope.

PAS, acid fuchsin orange G (AFOG) and methenamine silver (MET) staining of the patient kidney biopsy were performed at the Department of Pathology at the University Medical Center Freiburg.

**Immunofluorescence Staining**

Tissue sections were deparaffinized and rehydrated via xylol and a descending ethanol series. Heat-induced epitope retrieval was performed with citrate buffer pH 6.0 or TRIS/EDTA buffer pH 9.0 in a cooker. The slides were blocked with 5% BSA in 1x PBS for 1h at RT. Primary and secondary antibodies were incubated for 1h at RT. Images were taken with a Zeiss Axiovert M200 microscope equipped with an ApoTome.

**Podocyte morphometrical Analysis**

Immunofluorescence staining of paraffin-embedded mice kidney tissue was performed as described in the previous section. The tissues were stained with SYNPO as a podocyte cytoplasm marker, DACH1 as a podocyte nucleus marker, and DAPI as a general nucleus marker. Model-based stereology was applied to calculate glomerular volume, podocyte number, podocyte density, and average podocyte volume per glomerulus (1, 2). Average podocyte volume was calculated by dividing the calculated total podocyte cell volume

(TPCV) by the podocyte number (PN). 6 mice per group were analyzed and 20 glomeruli per section were randomly taken with the confocal microscope. Fiji imaging software (Max Planck Institute of Molecular Cell Biology and Genetics, Dresden, Germany) was used to measure the area of the glomerular tuft, podocyte nuclear number, and podocyte nuclear and cellular area in the sections. Each glomerulus was counted as a single observation and median values were used for statistical analysis and data presentation.

**Expansion Microscopy**

Tissue expansion

Tissue expansion for enhanced optical resolution in thin sections has previously been published and described in greater detail (ExPath) (3). Briefly and in analogy to the ExPath protocol, immunofluorescence stained tissue sections first underwent anchoring treatment with 0.1mg/ml Acryloyl-X (6-((acryloyl) amino) hexanoic acid, succinimidyl ester, (Thermo Fisher Scientific, Cat#A20770) at room temperature for 12h. The tissue sections were then embedded into a gelling solution consisting of 1xPBS, 2M NaCl, 8.625% sodium acrylate (Sigma Aldrich), 2.5% acrylamide (Sigma Aldrich), 0.1% N-N´-methylenbisacrylamide, 0.01% (Sigma Aldrich), 4-hydroxy-2,2,6,6-tetramethylpiperidin-1-oxyl (4HT, Sigma Aldrich), 0.2% TEMED and 0.2% APS. The tissue sections embedded in the gelling solution were then incubated at 4°C for 30 min, to allow for penetration of the gelling solution into the tissue. After that, gelling chambers, each consisting of two coverslips as spacers on either side of the tissue to prevent compression and the third coverslip on top of the tissue, were constructed around the tissue. The tissue sections were then incubated in a humidified oven at 37°C for 2h to complete gelation. Next, the gelling chambers were removed and the tissue sections were incubated in 8U/ml

proteinase K (Sigma-Aldrich, Cat# P2308-100MG) in a Tris/EDTA-based digestion buffer (50mM Tris (pH 8), 25 mM EDTA, 0.5% Triton X-100 and 0.8 M NaCl) at 60°C for 4h. The digested tissue sections were then removed from the slide and placed in doubly deionized water at room temperature for 60 min for isotropic expansion. After expansion, the tissue sections were removed from the doubly deionized water and mounted in glass-bottom chamber slides (Ibidi µ-Slide 2 Well Glass Bottom) for subsequent super-resolution imaging.

Imaging

Post-expansion super-resolution imaging was performed using a Zeiss LSM 800 confocal microscope with Airyscan using the optimized 63x objective and 8x digital zoom with subsequent Airyscan processing. Fiji imaging software (Max Planck Institute of Molecular Cell Biology and Genetics) was used to navigate the files and to adjust color balance.

**Transmission Electron microscopy**

TEM analysis was applied as previously described (4). In summary, tissues were fixed with 4% PFA and 1% glutaraldehyde and 1-2 $mm^3$ blocks of the kidney were cut. After dehydration, the tissues were embedded in epoxy resin (Durcupan ACM, Fluka, Sigma-Aldrich, Gillingham, UK). 40 nm ultrathin sections were cut and analyzed using a Phillips CM 100 transmission electron microscope. For quantitative analysis, 4 mice of each time point and genotype were analyzed. 15 to 20 random images of glomeruli were taken at a magnification of 2900x using a Phillips CM 100 transmission electron microscope. The Image J software were used to create an overlay using gridlines with a mesh size of 500nm randomly placed on top of the randomly taken images. Then, GBM thickness at

each crossing point of the grid lines were measured with the GBM. For Bowman capsule we did the same however here we measured only 4 to 7 randomly taken images per animal and the mesh size was 1µm. Determination of foot process width was performed as previously described and image analysis was done via ITEM software (Olympus) or ImageJ software (5).

**Construction and production of AAV vectors**

The human *P3H2* gene (accession number NM_018192.4) was amplified by PCR from a plasmid encoding *P3H2* cDNA (GenScript, Cat# OHu02725) with the forward primer AgeI: 5'-GGGACCGGTAACTTAAGCTTGGTACCGAG-3' and the reverse primer BsrGI: 5'-GGGTGTACATTATAGCTCATCTTTAGGGTTGATAT-3'. The AAV-CMV-eGFP plasmid was cloned by replacing the eGFP-Cre fragement in pAAV.CMV.HI.eGFP-Cre.WPRE.SV40 (Addgene Plasmid #105545) with eGFP only. The AAV-CMV-P3H2 plasmid was generated by subcloning the P3H2 cDNA into AgeI and BsrGI sites of AAV-CMV-eGFP plasmid.

Recombinant AAV vectors were produced by triple transfection of HEK293T/17 cells (ATCC CRL-11268) with AAV-shh10 (Plasmid #64867) encoding AAV2 rep and AAVshh10 cap, pxx6 helper plasmid (6), and the plasmid containing ITR-flanked transgene expression cassette. After three days of transfection, cells were harvested and lysed by three freeze-thaw cycles treated with Benzonase Nuclease (Sigma-Aldrich, Cat# E1014-25KU). AAV vectors were then purified by iodixanol gradient ultracentrifugation. Physical particles were quantified by real-time PCR with the forward primer: 5'-GGGACTTTCCTACTTGGCA-3' and the reverse primer: 5'-

GGCGGAGTTGTTACGACAT-3’ directed to the CMV promoter sequence, and titers are expressed as viral genomes per mL (vg/mL).

For overexpression of *P3H2* gene in immortalized podocyte cell lines, $1x10^6$ cells were seeded on 10 cm cell culture dish. After 24h, cells were infected with AAV vectors. $1x10^5$ vg/mL per cell AAV titer were used to infect the cells. After infection, differentiation of cells were started and ECM isolation was performed after differentiation.

**Adhesion assay**

150.000 cells per well were seeded on 24 well plates and incubated for 15 min at 37 °C incubator. After washing with 1x PBS, the cells were fixed with 4% PFA and stained with 0.1% Crystal Violet stain for 15 min at room temperature (RT). The membrane of the cells was permeabilized with 0.5% TritonX-100 for 30 min at RT. The absorbance of this solution was measured at 570 nM in a TECAN Sunrise Basic microplate reader.

**Migration assay**

20.000 cells per well were split into a culture-Insert 2 Well in µ-Dish 35 mm (Ibidi, Cat# 81176) and images were taken at 0h, 3h, 6h, and 9h. The cell-free area was measured by the ImageJ program to calculate the migrated area.

**Extracellular matrix isolation**

12 days differentiated *P3H2* KO and WT immortalized human podocyte cell lines were lysed with alkaline detergent buffer (20mM $NH_4OH$ and 0.5% v/v TritonX-100 in PBS) for 1 min at 37° C and washed with 1x PBS. After incubation with 10 µg/ml DNase I (Roche, Cat# 10104159001) for 1h at 37 °C, denuded ECM was scraped into reducing buffer

(50mM Tris-HCl pH 6.8, 10% w/v glycerol, 4% SDS, 8% 2-mercaptoethanol and 0.004% bromophenol blue).

**Glomerular basement membrane isolation**

This procedure to isolate GBM from glomeruli was adapted from Lennon et al., JASN, 2014 (7). Isolated mouse glomeruli were incubated with extraction buffer (10 mM Tris, 150 mM NaCl, 1% Triton X-100, 25 mM EDTA, 25 µg/ml leupeptin and aprotinin, 0.5 mM ABSFH for 30 min, and centrifuged at 14.000 x g for 10 min. The pellet was incubated for 30 min in an alkaline detergent buffer and centrifuged at 14.000 x g for 10 min. The pellet was incubated for 30 min in 10 mg/ml DNase I and centrifuged at 14.000 x g for 10 min. The final pellet was resuspended in reducing sample buffer to yield the ECM.

**Relative quantitative mass spectrometric analysis of the ECM and GBM proteome**

Isolated ECM and enriched GBM were prepared using the optimized single-pot, solid-phase-enhanced sample-preparation (SP3) protocol (8). In brief, 10 µg of ECM or GBM protein were taken per sample, and disulfide bonds reduced in the presence of 10 mM DTT at 60 °C for 30 min at RT. Cysteine residues were then alkylated in the presence of 20 mM iodoacetamide (IAA) at 37 °C for 30 min in the dark. 20 µg/µL of a 1:1 mix of carboxylate-modified paramagnetic beads (Sera-Mag Speed-Beads (Hydrophilic), and Sera-Mag Speed-Beads (Hydrophobic) were added to each sample. Acetonitrile (ACN) was added to a final concentration of 70% (v/v) ACN and samples were shaken at 900 x rpm at RT and then placed on a magnetic rack. The supernatant was removed, the beads were washed with 70% (v/v) ethanol, and then with 100% ACN. Beads were resuspended in 10 µL trypsin solution (1:50 Enzyme to protein ratio, 50 mM $NH_4HCO_3$) and samples

were digested at 37 °C overnight by shaking at 700 x rpm. After digestion, ACN was added, to a final concentration of 95% (v/v). After a brief incubation, samples were placed on a magnetic rack, the supernatant was removed, and beads were rinsed with 100% ACN. Peptides were eluted from the beads in 20 µL of a 2% (v/v) DMSO and transferred into a new tube. Samples were dried in a vacuum centrifuge.

**Analysis of the tryptic peptides with Liquid Chromatography coupled to Tandem Mass Spectrometry (LC-MS/MS) and Data Processing**

For LC-MS/MS analysis, samples were resuspended in 0.1% formic acid (FA) at a concentration of 1 µg/µl. LC-MS/MS measurements were performed on a Quadrupole Orbitrap hybrid mass spectrometer (Q Exactive, Thermo Fisher) coupled with a UPLC system (nanoAcquity, Waters). For analysis, 1 µg of peptides were loaded by autosampler injection onto a C18 reversed-phase (RP) trap column (Symmetry C18 trap column, 100 Å pore size, 5 µm particle diameters, 180 µm x 20 mm) and separated on a 20 cm C18 RP (Peptide BEH C18 column, 130 Å pore size, 1.7 µm particle diameters, 75 µm x 250 mm). Trapping was done for 5 min at a flow rate of 15 µl per min with 99% solvent A (0.1% FA) and 1% solvent B (0.1% FA in ACN). Separation and elution of peptides were achieved by a linear gradient from 1 to 30% solvent B in 60 min.

The eluting peptides were transferred in an Orbitrap Q Exactive mass spectrometer. MS1 scans were performed in positive mode over a scan range of 400-1300 m/z. The Orbitrap resolution was set to 70.000 with an AGC target of $1x10^6$ and a maximum injection time of 240 ms. Peptides with charge states between 2+ - 5+ above an intensity threshold of 100.000 were isolated with a 2 m/z isolation window in Top12 mode and fragmented with

a normalized collision energy of 28%. The fragments were measured with an Orbitrap resolution of 17.500, AGC target of $1x10^{5,}$ and 50 ms maximum injection time. Already fragmented peptides were excluded for 20 seconds.

The collected raw files were searched against the reviewed mouse protein database downloaded from Uniprot (release October 2019 with 17,013 protein sequences) processed with the Andromeda Algorithm included in the MaxQuant Software (Max Plank Institute for Biochemistry, Version 1.6.2.10). All samples were handled as individual experiments. The label-free quantification option with the match between runs was used. Trypsin was selected as an enzyme used to generate peptides, allowing a maximum of two missed cleavages. A minimal peptide length of 6 amino acids and maximal peptide mass of 6000 Da were defined. Oxidation of methionine and proline (hydroxylation), acetylation of protein N-termini, and the conversion of glutamine to pyro-glutamic acid were set as variable modifications. The carbamidomethylation of cysteines was selected as a fixed modification. The error tolerance for the first precursor search was 20 ppm, for the following main search 4.5 ppm. Fragment spectra were matched with 20 ppm error tolerance. The false discovery rate for peptide spectrum matches and proteins was set to 1%. For Quantification, all identified razor and unique peptides were considered.

The ProteinGroups.txt result files from MaxQuant were loaded into Perseus software (Max Plank Institute for Biochemistry, Version 1.5.8.5). The quantitative LFQ Intensity values for protein groups were used as main columns. The quantitative values for all protein groups were transformed into log2 values and normalized by the median. Hierarchical clustering, student’s t-test, and principal component analysis (PCA) were performed.

**Statistical data analysis**

The data in the diagrams of the results section are shown in different types of plots. All statistical analysis were performed and plots were prepared by using GraphPad Prism (v8.4.0). The data are presented as mean with SD or median with IQR. Two-tailed Student's t-test and Mann Whitney's U test were used to test for significance between experimental and control group. When three or more groups were assessed, one-way ANOVA with Tukey multiple comparison post hoc tests was used. A p-value of $p < 0.05$ (*) was set as the significance level. A p-value of $p < 0.01$ (**) was found as very significant and a p-value of $p < 0.001$ (***) and $p < 0.0001$ (****) were found as highly significant.

## References


1. Puelles VG, Bertram JF, and Moeller MJ. Quantifying podocyte depletion: theoretical and practical considerations. *Cell Tissue Res.* 2017;369(1):229-36.

2. Puelles VG, van der Wolde JW, Wanner N, Scheppach MW, Cullen-McEwen LA, Bork T, et al. mTOR-mediated podocyte hypertrophy regulates glomerular integrity in mice and humans. *JCI Insight.* 2019;4(18).

3. Zhao Y, Bucur O, Irshad H, Chen F, Weins A, Stancu AL, et al. Nanoscale imaging of clinical specimens using pathology-optimized expansion microscopy. *Nat Biotechnol.* 2017;35(8):757-64.

4. Brinkkoetter PT, Bork T, Salou S, Liang W, Mizi A, Ozel C, et al. Anaerobic Glycolysis Maintains the Glomerular Filtration Barrier Independent of Mitochondrial Metabolism and Dynamics. *Cell Rep.* 2019;27(5):1551-66 e5.

5. Bechtel W, Helmstadter M, Balica J, Hartleben B, Kiefer B, Hrnjic F, et al. Vps34 deficiency reveals the importance of endocytosis for podocyte homeostasis. *J Am Soc Nephrol.* 2013;24(5):727-43.

6. Xiao X, Li J, and Samulski RJ. Production of high-titer recombinant adeno-associated virus vectors in the absence of helper adenovirus. *J Virol.* 1998;72(3):2224-32.

7. Lennon R, Byron A, Humphries JD, Randles MJ, Carisey A, Murphy S, et al. Global analysis reveals the complexity of the human glomerular extracellular matrix. *J Am Soc Nephrol.* 2014;25(5):939-51.

8. Sielaff M, Kuharev J, Bohn T, Hahlbrock J, Bopp T, Tenzer S, et al. Evaluation of FASP, SP3, and iST Protocols for Proteomic Sample Preparation in the Low Microgram Range. *J Proteome Res.* 2017;16(11):4060-72.
